# Prescribing of antipsychotics among people with recorded personality disorder in primary care: a retrospective nationwide cohort study using The Health Improvement Network primary care database

**DOI:** 10.1136/bmjopen-2021-053943

**Published:** 2022-03-07

**Authors:** Sarah Hardoon, Joseph Hayes, Essi Viding, Eamon McCrory, Kate Walters, David Osborn

**Affiliations:** 1 Division of Psychiatry, UCL, London, UK; 2 Division of Psychology and Language Sciences, UCL, London, UK; 3 Department of Primary Care and Population Health, UCL, London, UK

**Keywords:** personality disorders, primary care, therapeutics

## Abstract

**Objectives:**

To investigate the extent of antipsychotic prescribing to people with recorded personality disorder (PD) in UK primary care and factors associated with such prescribing.

**Design:**

Retrospective cohort study.

**Setting:**

General practices contributing to The Health Improvement Network UK-wide primary care database, 1 January 2000–31 December 2016.

**Participants:**

46 210 people registered with participating general practices who had a record of PD in their general practice notes. 1358 (2.9%) people with missing deprivation information were excluded from regression analyses; no other missing data.

**Main outcome measures:**

Prescriptions for antipsychotics in general practice records and length of time in receipt of antipsychotic prescriptions.

**Results:**

Of 46 210 people with recorded PD, 15 562 (34%) were ever prescribed antipsychotics. Among the subgroup of 36 875 people with recorded PD, but no recorded severe mental illness (SMI), 9208 (25%) were prescribed antipsychotics; prescribing was lower in less deprived areas (adjusted rate ratio (aRR) comparing least to most deprived quintile: 0.56, 95% CI 0.48 to 0.66, p<0.001), was higher in females (aRR:1.25, 95% CI 1.16 to 1.34, p<0.001) and with a history of adverse childhood experiences (aRR:1.44, 95% CI 1.28 to 1.56, p<0.001). Median time prescribed antipsychotics was 605 days (IQR 197–1639 days). Prescribing frequency has increased over time.

**Conclusions:**

Contrary to current UK guidelines, antipsychotics are frequently and increasingly prescribed for extended periods to people with recorded PD, but with no history of SMI. An urgent review of clinical practice is warranted, including the effectiveness of such prescribing and the need to monitor for adverse effects, including metabolic complications.

**Strengths and limitations of this study**

- In the present report, we identified a very large UK-wide cohort of >46 000 people with a recorded personality disorder (PD) diagnosis, enabling, for the first time, a detailed UK-wide exploration of prescribing practices in PD over an 18-year period from 2000 to 2016.
- The cohort was derived from The Health Improvement Network primary care database which has been shown to be socially and demographically representative of wider UK general practice.
- The study benefits from complete records on the outcome (antipsychotics prescribed in primary care, including the type of antipsychotic), as all prescriptions are automatically computerised.
- Potential limitations include limited information on alternative treatment options, such as psychological therapy, and that it is not possible to ascertain how the PD diagnoses were arrived at or to verify them.

## Introduction

The core features of personality disorder (PD) diagnoses are pervasive patterns of behaviour and maladaptive traits leading to substantial personal distress, social dysfunction and disruption to others, often beginning in early adult life.^1^ Prevalence of PD in the community is estimated to be 6%–10%, but estimates for PD vary globally and by clinical setting,^2^ with limited research regarding the prevalence in primary care settings.^3^ There is limited evidence on the effectiveness of antipsychotic pharmacotherapy in PD. Results from randomised controlled trials mainly focus on emotionally unstable PD (EUPD) populations, but the findings are limited by short-term follow-up, small sample sizes and variability in outcome measures.^4^ In the UK, treatment guidelines (from the National Institute for Health and Care Excellence (NICE)) exist for EUPD and dissocial PD. For both diagnoses it is recommended that ‘drug treatment should not be used’, apart from short-term sedating medication during crises in EUPD.^5^ This is echoed by guidelines for EUPD from Australia’s National Health and Medical Research Council.^6^ Conversely, the American Psychiatric Association recommends targeted pharmacotherapy for PD: antidepressant medication for affective instability, mood stabilisers for impulsive aggression and antipsychotics for cognitive-perceptive disturbance.^7^ Cochrane reviews find no evidence for the efficacy of antidepressant medication, but some evidence that mood stabilisers may reduce affective dysregulation and impulsive–aggression and that antipsychotic medication may improve cognitive–perceptual symptoms and affective dysregulation in PD.^8^ Despite this mixed evidence, and counter to the NICE guidelines, there is some limited data suggesting that psychotropic prescribing for PD is occurring in the UK mental health service setting.^9^ There is an urgent need to ascertain the wider extent and duration of psychotropic prescribing in PD. There is a particular concern regarding the prescribing of antipsychotics given their adverse event profile, including metabolic and cardiovascular risks.^10^ Further, the characteristics of individuals prescribed psychotropic medications need to be determined to understand who may be most likely to receive these medications. For example, females are more likely than males to be diagnosed with borderline or EUPD and there is a high prevalence of adverse childhood experiences among those with EUPD.^11^ Yet it is not known whether patients of particular gender, PD diagnosis or life history are more likely to receive antipsychotic medication. Time trends in antipsychotic prescribing for PD have not been examined and may be important, as UK mental health services and social care provision have been negatively affected by economic austerity.^12^ This study aimed to determine how frequently antipsychotics are prescribed for people with a diagnosis of PD in UK primary care as well as factors associated with this prescribing. The three main aims of the study were: (1) To quantify the prevalence and duration of antipsychotic prescribing among patients with a record of PD, particularly those without any diagnostic record of severe mental illness (SMI); (2) To determine whether gender, age, area level deprivation, mental health comorbidities, category of PD diagnosis or a recorded history of adverse childhood experiences affect the likelihood of being prescribed antipsychotics and (3) To examine time trends in prescribing of antipsychotics.

## Methods

### Study design

Retrospective nationwide cohort study.

### Data source

We used data from The Health Improvement Network (THIN) primary care database, which is a clinical database comprising, at time of analysis, computerised anonymised patient records retrieved from 744 member general practices across the UK, corresponding to approximately 6% of the UK population.^13^ Patient records are longitudinal, including all information recorded by the general practitioner (GP) from when the patient registered with the member practice until the date the practice last contributed data to THIN or the patient left the practice. Records for a patient are linked via an anonymised patient identifier.

The database includes all diagnoses, symptoms and other health data (blood tests, health indicators) recorded by the GP in the patient’s computerised records, as well as all prescriptions issued. Diagnoses, symptoms and other health information in THIN are coded using the Read code clinical classification system.^14^ The database has been shown to be socially and demographically representative of the UK general practice population.^13^

### Study population

From the THIN database population, we identified the smaller study population (our PD cohort) comprising people registered with a participating THIN general practice for at least some part of the period 2000–2016, who had a record in their general practice notes at any time during or prior to this period of a diagnosis of PD (specific or non-specific), identified via the presence of a Read code indicative of a PD diagnosis. A list of relevant Read codes was developed using established search techniques^15^ and with reference to International Classification of Diseases (ICD-10) chapters F60–F61. The diagnostic list was verified by a clinician (JH). The complete Read code list is provided in online supplemental table 1. All permanently registered patients were included from the date the general practice reached previously defined, established data quality thresholds: acceptable mortality rate date^16^ and acceptable computer usage date.^17^

10.1136/bmjopen-2021-053943.supp1 Supplementary data

PD Read codes were categorised in line with current versions of the ICD-10 and the Diagnostic and Statistical Manual of Mental Disorders-5 as: paranoid, schizoid, dissocial, EUPD (including borderline), histrionic, anankastic, anxious/avoidant, dependent and other (chiefly comprising codes for non-specific PD, but also including diagnoses such as eccentric, Haltlose, immature, narcissistic, masochistic, passive-aggressive and mixed). Where an individual had more than one type of specific PD record, the most recent record was assigned as their diagnosis.

### Outcomes

The primary outcome was a prescription for antipsychotic medication in the individual’s primary care records, any time after the initial PD diagnosis. We included all first generation and second generation antipsychotics in British National Formulary chapters 4.2.1; 4.2.2 (listed in online supplemental table 2).

It is not possible to ascertain from THIN records whether a prescription that was issued was actually dispensed or used by the individual. Therefore, we also included an outcome of at least two consecutive prescriptions being issued fewer than 84 days apart as a likely indication that the first prescription had been used (corresponds to 3×28 days, a common period for which longer-term medicines are issued in UK primary care).

We also determined the length in days of continuous antipsychotic treatment. A continuous treatment period was defined as repeat prescriptions for antipsychotics no more than 84 days apart. The length of a continuous treatment period was then calculated from the date of the first prescription in the period to date of last prescription +84 days.

### Follow-up period

PD cohort entry was defined as the latest date of: registration at the general practice, the established practice acceptable mortality rate date,^16^ the established practice acceptable computer usage date,^17^ date of first PD diagnosis record, or 1 January 2000. End of follow-up for an individual was the earliest of: date of death, date patient transferred out of practice, latest date practice contributed data to THIN, or 31 December 2016.

### Covariates

Antipsychotic prescribing was explored according to the following sociodemographic characteristics: gender, age and Townsend score for area-level social deprivation for the area (postcode) in which the individual lived (grouped into quintiles).^18^ The Townsend score incorporates four variables: percentage of adults unemployed, percentages non-car ownership, percentage non-home ownership and percentage overcrowded households.

We extracted data regarding comorbid mental health conditions defined as: (SMI, including schizophrenia, bipolar disorder and other non-organic psychotic illness),^19^ depression, anxiety, phobia, hypochondria, obsessive–compulsive disorder, other neurosis, eating disorder, post-traumatic stress disorder. Each of these conditions were identified by the presence of a Read code at any time, using lists of Read codes developed through established techniques, with reference to relevant ICD chapters and verified by clinician (JH).

The presence of recorded adverse childhood experiences was identified from records with Read codes specifically relating to: contact with social services, child protection procedures, child on at risk register, child-at-risk case conference, out-of-home care or codes corresponding to items in the Centers for Disease Control and Prevention short adverse childhood experiences tool,^20^ that is; direct reference to childhood maltreatment (physical or emotional abuse or neglect), or direct reference to household dysfunction (such as parental substance misuse). Codes included were either (1) codes recorded in childhood (when individual <17 years) or (2) codes recorded during adulthood indicative of a history of the above in childhood. The code list was based on previous work examining recording of childhood maltreatment in THIN.^21^

### Patient and public involvement

A group of service users was established in conjunction with the McPin Foundation, called the Pathfinder Data Science Patient and Public Involvement Group. Through regular meetings over the course of the study, members of the group were involved in the design, conduct and reporting plans for our research.

### Statistical analysis

We first determined the percentages of people: (1) receiving at least one antipsychotic prescription and (2) at least two antipsychotic prescriptions fewer than 84 days apart. We also determined percentages of people with a continuous treatment periods of at least (1) 6 months, (2) 1 year, (3) 3 years and (4) 5 years. Percentages were computed for the whole sample and then by: gender, age (in 10-year age bands), Townsend deprivation quintile, diagnostic category of PD, recorded mental health comorbidity and whether an individual had a recorded history of adverse childhood experiences. Analyses were stratified by whether patients ever had an additional recorded diagnosis of SMI. For the continuous treatment periods percentages calculations, the study sample was restricted to only individuals who had at least that length of follow-up in the database (6 months, 1 year, 3 years or 5 years).

We then used univariable and multivariable Poisson regression, with follow-up time for individuals as an offset, to estimate rate ratios for antipsychotic prescribing, to take account of differing follow-up times for individuals in the study. Rates were computed as total people receiving a prescription/ total follow-up time for people in each covariate category. Regressions were stratified by ever receiving a diagnosis of SMI. Multilevel Poisson models were used, with individuals nested in general practices, to account for potential clustering of individuals in general practices and clustering of prescribing practices within general practices.

Time trends in antipsychotic prescribing were determined as yearly prevalence estimates of antipsychotic prescriptions: namely the number within the sample with at least one antipsychotic prescription in that year/number of the sample present in the database that year.

We calculated the median and IQR of length of longest continuous treatment period for all antipsychotics and for individual agents. The numbers of individuals receiving at least one prescription for individual agents were also computed.

We carried out two sensitivity analyses using different definitions of adverse childhood experiences. In the first sensitivity analysis, we restricted the definition of adverse childhood experiences to a record (current or retrospective) of contact with social services before age 17 years. In the second sensitivity analysis, we only included people with primary care data available from childhood (from at least age 11 years), to enable capture of adverse childhood experiences as recorded during childhood, rather than historically.

Stata (V.14) was used for all analyses.

## Results

The Personality Disorder cohort comprised 46 210 people who had a record of PD in their general practice notes. Median duration in the study was 3.2 years (IQR 1.1–8.0 years). Just over half the PD cohort were female (23 528, 51%), and the average age at study entry was 43 years (table 1). The PD cohort had greater levels of deprivation relative to the general population, with 11 928 (26%) of people living in areas within the most deprived quintile, and 5523 (12%) in the least deprived quintile. The most commonly recorded diagnostic category of PD was EUPD recorded among 12 300 people (27% of the PD cohort), followed by anankastic (3648, 8%). However, 20 147 (44%) of the PD cohort had only non-specific PD records including primarily 14 321 (31%) with Read code E21.00 ‘PD’ and 4676 (10%) with Read code E21z.00 ‘PD NOS (Not Otherwise Specified)’ (online supplemental table 1). A record of ever being diagnosed with an SMI was present in 9335 people (20% of the PD cohort), while 34 522 (75%) and 23 595 (51%) had records of depression and anxiety respectively. A history of adverse childhood experiences was recorded for 4742 people (10%) in the PD cohort.

**Table 1:** Baseline characteristics of study cohort

	All (n=46 210)	Recorded SMI (n=9335)	No recorded SMI (n=36 875)
	Mean (SD)	Mean (SD)	Mean (SD)
Age at study entry*, years	42.65 (16.39)	43.37 (15.24)	42.47 (16.66)
	**N (%)**	**N (%)**	**N (%)**
Gender			
Men	22 682 (49.1)	4689 (50.2)	17 993 (48.8)
Women	23 528 (50.9)	4646 (49.8)	18 882 (51.2)
Age, years			
0–15	384 (0.8)	6 (0.1)	378 (1.0)
15–19	1729 (3.7)	179 (1.9)	1550 (4.2)
20–24	4482 (9.7)	707 (7.6)	3775 (10.2)
25–29	5089 (11.0)	1004 (10.8)	4085 (11.1)
30–34	5312 (11.5)	1178 (12.6)	4134 (11.2)
35–39	5541 (12.0)	1291 (13.8)	4250 (11.5)
40–44	5251 (11.4)	1214 (13.0)	4037 (10.9)
45–49	4379 (9.5)	1002 (10.7)	3377 (9.2)
50–54	3802 (8.2)	783 (8.4)	3019 (8.2)
55–59	3053 (6.6)	596 (6.4)	2457 (6.7)
60–64	2335 (5.1)	469 (5.0)	1866 (5.1)
65–69	1657 (3.6)	311 (3.3)	1346 (3.7)
70–74	1152 (2.5)	214 (2.3)	938 (2.5)
75–100	2044 (4.4)	381 (4.1)	1663 (4.5)
Townsend deprivation score			
1 (least deprived)	5523 (12.0)	893 (9.6)	4630 (12.6)
2	6700 (14.5)	1144 (12.3)	5556 (15.1)
3	9047 (19.6)	1762 (18.9)	7285 (19.8)
4	11 654 (25.2)	2563 (27.5)	9091 (24.7)
5 (most deprived)	11 928 (25.8)	2693 (28.8)	9235 (25.0)
Missing	*1358* (*2.9*)	*280* (*3*)	*1078* (*2.9*)
ICD-10 diagnostic category of PD			
Paranoid	1988 (4.3)	981 (10.5)	1007 (2.7)
Schizoid	1346 (2.9)	565 (6.1)	781 (2.1)
Dissocial	2407 (5.2)	536 (5.7)	1871 (5.1)
Emotionally unstable	12 300 (26.6)	2591 (27.8)	9709 (26.3)
Histrionic	1168 (2.5)	178 (1.9)	990 (2.7)
Anankastic	3648 (7.9)	347 (3.7)	3301 (9.0)
Anxious	501 (1.1)	62 (0.7)	439 (1.2)
Dependent	2705 (5.9)	411 (4.4)	2294 (6.2)
Other*	20 147 (43.6)	3664 (39.3)	16 483 (44.7)
Recorded psychiatric comorbidity			
Any SMI	9335 (20.2)	9335 (100)	0 (0)
Schizophrenia	*4105* (*8.9*)	*4105* (*44.0*)	*0* (*0*)
Bipolar disorder	*2987* (*6.5*)	*2987* (*32.0*)	*0* (*0*)
Other psychotic illness	*5071* (*11.0*)	*5071* (*54.3*)	*0* (*0*)
Post-traumatic stress disorder	1468 (3.2)	306 (3.3)	1162 (3.2)
Eating disorder	2852 (6.2)	613 (6.6)	2239 (6.1)
Depression	34 522 (74.7)	7116 (76.2)	27 406 (74.3)
Anxiety	23 595 (51.1)	5006 (53.6)	18 589 (50.4)
Phobia	3279 (7.10)	666 (7.1)	2613 (7.1)
Hypochondria	416 (0.9)	117 (1.3)	299 (0.8)
Obsessive–compulsive disorder	5333 (11.5)	831 (8.9)	4502 (12.2)
Other neurosis	1353 (2.9)	332 (3.6)	1021 (2.8)
Recorded adverse childhood experience			
No record	41 468 (89.7)	8389 (89.9)	33 079 (89.7)
Record	4742 (10.3)	946 (10.1)	3796 (10.3)

*Other PD includes: eccentric, Haltlose, immature, narcissistic, masochistic, passive-aggressive, mixed and non-specific.

ICD, International Classification of Diseases; PD, personality disorder; SMI, severe mental illness.

### Prevalence of antipsychotic prescribing

One-third of the PD cohort of people with recorded diagnosis of PD were prescribed antipsychotics at least once after their first PD record (15 562, 34%, table 2). The majority of those prescribed antipsychotics (9208, 59%) in the PD cohort had no record of SMI to explain the prescribing. Indeed, restricting the PD cohort to 36 875 individuals with recorded PD but no record of SMI, one quarter (9208, 25%) had been prescribed antipsychotics

**Table 2:** Proportions of individuals receiving at least one prescription for antipsychotics during follow-up

	Total	Prescribed antipsychotics, n (%)		Recorded SMI	Prescribed antipsychotics, n (%)		No recorded SMI	Prescribed antipsychotics, n (%)	
All	46 210	15 562	(33.7)	9335	6354	(68.1)	36 875	9208	(25.0)
Gender									
Men	22 682	6958	(30.7)	4689	3044	(64.9)	17 993	3914	(21.8)
Women	23 528	8604	(36.6)	4646	3310	(71.2)	18 882	5294	(28.0)
Age, years									
0–15	384	26	(6.8)	6	4	(66.7)	378	22	(5.8)
15–19	1729	556	(32.2)	179	127	(70.9)	1550	429	(27.7)
20–24	4482	1543	(34.4)	707	450	(63.6)	3775	1093	(29.0)
25–29	5089	1866	(36.7)	1004	665	(66.2)	4085	1201	(29.4)
30–34	5312	2009	(37.8)	1178	835	(70.9)	4134	1174	(28.4)
35–39	5541	2068	(37.3)	1291	905	(70.1)	4250	1163	(27.4)
40–44	5251	1924	(36.6)	1214	840	(69.2)	4037	1084	(26.9)
45–49	4379	1503	(34.3)	1002	718	(71.7)	3377	785	(23.2)
50–54	3802	1169	(30.7)	783	524	(66.9)	3019	645	(21.4)
55–59	3053	816	(26.7)	596	421	(70.6)	2457	395	(16.1)
60–64	2335	586	(25.1)	469	308	(65.7)	1866	278	(14.9)
65–69	1657	420	(25.3)	311	190	(61.1)	1346	230	(17.1)
70–74	1152	337	(29.3)	214	128	(59.8)	938	209	(22.3)
75–100	2044	739	(36.2)	381	239	(62.7)	1663	500	(30.1)
Townsend deprivation score									
1 (least deprived)	5523	1486	(26.9)	893	592	(66.3)	4630	894	(19.3)
2	6700	1932	(28.8)	1144	772	(67.5)	5556	1160	(20.9)
3	9047	2956	(32.7)	1762	1204	(68.3)	7285	1752	(24.0)
4	11 654	4176	(35.8)	2563	1751	(68.3)	9091	2425	(26.7)
5 (most deprived)	11 928	4559	(38.2)	2693	1861	(69.1)	9235	2698	(29.2)
Missing	*1358*	*453*	(*33.4*)	*280*	*174*	(*62.1*)	*1078*	*279*	(*25.9*)
ICD-10 diagnostic category of PD									
Paranoid	1988	1026	(51.6)	981	693	(70.6)	1007	333	(33.1)
Schizoid	1346	536	(39.8)	565	394	(69.7)	781	142	(18.2)
Dissocial	2407	763	(31.7)	536	337	(62.9)	1871	426	(22.8)
Emotionally unstable	12 300	5573	(45.3)	2591	1850	(71.4)	9709	3723	(38.3)
Histrionic	1168	285	(24.4)	178	116	(65.2)	990	169	(17.1)
Anankastic	3648	700	(19.2)	347	223	(64.3)	3301	477	(14.5)
Anxious	501	130	(25.9)	62	42	(67.7)	439	88	(20.0)
Dependent	2705	686	(25.4)	411	303	(73.7)	2294	383	(16.7)
Other	20 147	5863	(29.1)	3664	2396	(65.4)	16 483	3467	(21.0)
Psychiatric comorbidity									
Any SMI	9335	6354	(68.1)	9335	6354	(68.1)	0	0	(.)
Schizophrenia	4105	2981	(72.6)	4105	2981	(72.6)	0	0	(.)
Bipolar disorder	2987	2044	(68.4)	2987	2044	(68.4)	0	0	(.)
Other psychotic illness	5071	3583	(70.7)	5071	3583	(70.7)	0	0	(.)
Post-traumatic stress disorder	1468	731	(49.8)	306	223	(72.9)	1162	508	(43.7)
Eating disorder	2852	1233	(43.2)	613	448	(73.1)	2239	785	(35.1)
Depression	34 522	12 698	(36.8)	7116	4979	(70.0)	27 406	7719	(28.2)
Anxiety	23 595	9201	(39.0)	5006	3601	(71.9)	18 589	5600	(30.1)
Phobia	3279	1272	(38.8)	666	470	(70.6)	2613	802	(30.7)
Hypochondria	416	176	(42.3)	117	81	(69.2)	299	95	(31.8)
Obsessive–compulsive disorder	5333	1517	(28.4)	831	560	(67.4)	4502	957	(21.3)
Other neurosis	1353	466	(34.4)	332	245	(73.8)	1021	221	(21.6)
No recorded psychiatric comorbidity	5763	874	(15.2)	0	0	(.)	5763	874	(15.2)
Recorded adverse childhood experiences									
No record	41 468	13 798	(33.3)	8389	5774	(68.8)	33 079	8024	(24.3)
Record	4742	1764	(37.2)	946	580	(61.3)	3796	1184	(31.2)

ICD-10, International Classification of Diseases-10th revision; PD, personality disorder; SMI, severe mental illness.

Percentages of people in the PD cohort prescribed antipsychotics by gender, age, deprivation, mental health comorbidity, type of PD and recorded history of adverse childhood experiences, are shown in table 2. Among those with recorded PD but without recorded SMI, prescribing of antipsychotics was higher among females than males (5294 out of 18 882, 28% vs 3914 out of 17 993, 22%) and highest among those aged 20–34 years and over 75 years, compared with other age groups. Prescribing increased with deprivation, from 19% (894 out of 4630) in the least deprived quintile to 29% (2698 out of 9235) in the most deprived quintile. There was some variation in prescribing according to diagnostic category of PD, with prescribing highest among those with record of EUPD (3723 out of 9709, 38%), followed by paranoid PD (333 out of 1007, 33%). Those who had a recorded history of adverse childhood experiences in their GP record were more likely to be prescribed antipsychotics than those who did not (1184 out of 3796, 31% vs 8024 out of 33 079, 24%).

Adjusted and unadjusted rate ratios (aRR) comparing rates of prescribing of antipsychotics for different levels of the sociodemographic characteristics, are shown in table 3. The differences in prescribing of antipsychotics by gender, age, and deprivation, type of PD and record of adverse childhood experience all remained significant after adjustment in the group with no additional record of SMI. The aRR comparing women to men was 1.25 (95% CI 1.16 to 1.34, p<0.001). The aRR comparing those over 75 years to those aged 35–44 years was 2.02 (95% CI 1.75 to 2.34, p<0.001). The aRR comparing the least to most deprived areas was 0.56 (95% CI 0.48 to 0.66, p<0.001). Rates for all diagnostic categories of PD were statistically significantly lower than for EUPD with aRRs relative to EUPD of between 0.19 (95% CI 0.15 to 0.23, p<0.001) for anankastic PD and 0.63 (95% CI 0.53 to 0.74, p<0.001) for paranoid PD. The aRR comparing history to no history of adverse childhood experiences was 1.41 (95% CI 1.28 to 1.56, p<0.001).

**Table 3:** Rate ratios for receiving at least one antipsychotic prescription any time after first PD record

Predictor	Recorded SMI				No recorded SMI			
	Univariable models		Multivariable models*		Univariable models		Multivariable models*	
	RR (95% CI)	P value	RR (95% CI)	P value	RR (95% CI)	P value	RR (95% CI)	P value
Gender								
Men	1		1		1		1	
Women	1.29 (1.15 to 1.45)	<0.001	1.17 (1.04 to 1.32)	0.01	1.47 (1.34 to 1.61)	<0.001	1.25 (1.16 to 1.34)	<0.001
Age, years								
0–15	0.2 (0.05 to 0.72)	0.01	0.18 (0.05 to 0.58)	0.004	0.12 (0.08 to 0.20)	<0.001	0.14 (0.08 to 0.22)	<0.001
15–24	1.03 (0.85 to 1.25)	0.8	0.89 (0.73 to 1.08)	0.2	1.63 (1.45 to 1.83)	<0.001	1.14 (1.01 to 1.30)	0.04
25–34	1.21 (1.03 to 1.43)	0.02	1.13 (0.96 to 1.33)	0.1	1.38 (1.28 to 1.50)	<0.001	1.17 (1.06 to 1.30)	0.002
35–44	1		1		1		1	
45–54	0.99 (0.84 to 1.17)	0.9	1.06 (0.89 to 1.25)	0.5	0.68 (0.62 to 0.73)	<0.001	0.78 (0.71 to 0.85)	<0.001
55–64	0.77 (0.63 to 0.95)	0.01	0.86 (0.70 to 1.06)	0.2	0.41 (0.36 to 0.46)	<0.001	0.53 (0.45 to 0.61)	<0.001
65–74	0.68 (0.52 to 0.89)	0.004	0.75 (0.57 to 0.98)	0.04	0.55 (0.47 to 0.65)	<0.001	0.74 (0.62 to 0.89)	0.001
75–100	0.99 (0.76 to 1.29)	0.9	1.1 (0.84 to 1.44)	0.5	1.52 (1.31 to 1.75)	<0.001	2.02 (1.75 to 2.34)	<0.001
		<0.001†		0.003†		<0.001†		<0.001†
Townsend deprivation score								
1 (least deprived)	0.75 (0.60 to 0.93)	0.01	0.79 (0.63 to 0.98)	0.03	0.46 (0.38 to 0.56)	<0.001	0.56 (0.48 to 0.66)	<0.001
2	0.83 (0.67 to 1.03)	0.09	0.88 (0.71 to 1.08)	0.2	0.53 (0.47 to 0.60)	<0.001	0.64 (0.57 to 0.71)	<0.001
3	0.85 (0.72 to 1.00)	0.06	0.87 (0.74 to 1.03)	0.1	0.7 (0.63 to 0.76)	<0.001	0.78 (0.71 to 0.84)	<0.001
4	0.89 (0.78 to 1.03)	0.1	0.89 (0.78 to 1.02)	0.1	0.87 (0.80 to 0.94)	0.001	0.9 (0.83 to 0.97)	0.007
5 (most deprived)	1		1		1		1	
		0.08†		0.2†		<0.001†		<0.001†
ICD-10 diagnostic category of PD								
Anankastic	0.32 (0.23 to 0.44)	<0.001	0.35 (0.26 to 0.48)	<0.001	0.14 (0.12 to 0.16)	<0.001	0.19 (0.16 to 0.21)	<0.001
Anxious	0.44 (0.25 to 0.80)	0.006	0.47 (0.26 to 0.85)	0.01	0.23 (0.15 to 0.35)	<0.001	0.29 (0.20 to 0.41)	<0.001
Emotionally unstable	1		1		1		1	
Dependent	0.57 (0.41 to 0.78)	0.001	0.62 (0.44 to 0.86)	0.004	0.17 (0.14 to 0.21)	<0.001	0.22 (0.17 to 0.27)	<0.001
Dissocial	0.6 (0.46 to 0.80)	<0.001	0.66 (0.49 to 0.88)	0.005	0.34 (0.28 to 0.40)	<0.001	0.45 (0.39 to 0.52)	<0.001
Histrionic	0.35 (0.24 to 0.51)	<0.001	0.37 (0.25 to 0.54)	<0.001	0.15 (0.13 to 0.19)	<0.001	0.19 (0.15 to 0.23)	<0.001
Other	0.54 (0.47 to 0.62)	<0.001	0.57 (0.49 to 0.67)	<0.001	0.27 (0.25 to 0.30)	<0.001	0.33 (0.30 to 0.36)	<0.001
Paranoid	0.68 (0.55 to 0.83)	<0.001	0.72 (0.57 to 0.91)	0.005	0.52 (0.44 to 0.61)	<0.001	0.63 (0.53 to 0.74)	<0.001
Schizoid	0.42 (0.32 to 0.53)	<0.001	0.46 (0.35 to 0.60)	<0.001	0.18 (0.14 to 0.23)	<0.001	0.24 (0.18 to 0.33)	<0.001
		<0.001†		<0.001†		<0.001†		<0.001†
Recorded adverse childhood experiences								
No record	1		1		1		1	
Record	1.1 (0.92 to 1.31)	0.3	0.93 (0.78 to 1.10)	0.4	2.32 (2.03 to 2.65)	<0.001	1.41 (1.28 to 1.56)	<0.001

From Poisson regression, with generalised estimating equations to account for potential clustering of patients in general practices.

*Adjusted for all the other characteristics considered.

†P values in italics indicate significance of categorical variables as a whole, as opposed to the individual categories.

ICD-10, International Classification of Diseases-10th revision; PD, personality disorder.

### Time trends

**Figure 1:**
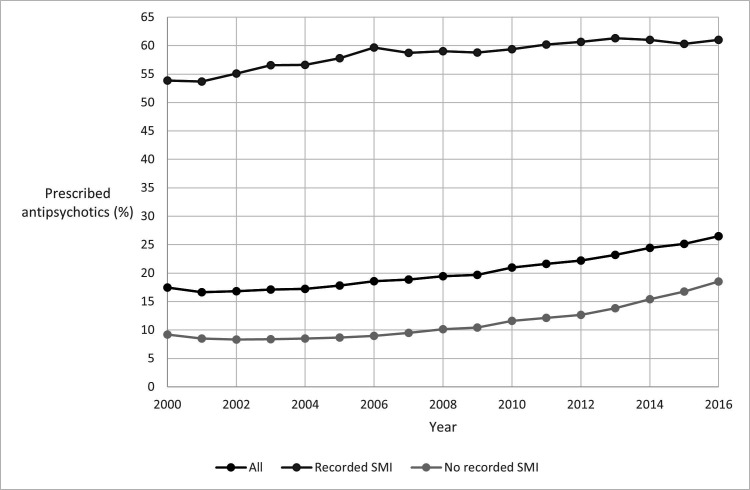
Percentage of cohort prescribed antipsychotics in each calendar year*. *Percentage of cohort prescribed antipsychotics in each calendar year=100 × (number of people from cohort present in that calendar year with at least one antipsychotic prescription in that year)/(number of people from cohort present in that calendar year). SMI, severe mental illness.

Figure 1 presents for each calendar year, the percentage of people in the PD cohort prescribed at least one antipsychotic in that year. The percentage of people receiving prescriptions increased over time: Of 6465 individuals who were present in the PD cohort in 2000, 1129 (17%) received antipsychotics in that year. Of 10 399 individuals present in the PD cohort in 2016, 2755 (26%) received antipsychotics (figure 1). Among those without a record of SMI, prescribing increased from 9% (484 out of 5268) in 2000 to 19% (1567 out of 8451) in 2016.

### Duration of antipsychotic treatment in PD

The median duration of continuous prescriptions of antipsychotics was 605 days (IQR 197–1639 days). Among those with no recorded SMI, the median duration of continuous treatment was 439 days (IQR 150–1218 days). For each of these results, we only assessed the longest period of treatment for each individual.

Among the total PD cohort of 46 210 people, 13 898 (30%) received at least two consecutive antipsychotic prescriptions within 84 days, and substantial numbers were prescribed antipsychotics over at least 6 months (11 906 out of 40 850 present for at least 6 months, 29%), 1 year (9588 out of 35 794, 27%), 3 years (5492 out of 24 081, 23%) and 5 years (3529 out of 17 588, 20%) (online supplemental table 3). Among the subgroup of individuals without recorded SMI, 22% (7925 out of 36 875) received at least two consecutive prescriptions within 84 days, and many were prescribed antipsychotics for more than 6 months (6548 out of 32 569, 20%), 1 year (4997 out of 28 576, 18%), 3 years (2538 out of 19 340, 13%) and 5 years (1485 out of 14 203, 11%).

### Individual antipsychotics agents

The most commonly prescribed antipsychotics in the PD cohort were Quetiapine (prescribed to 37%; 5819 of 15 562 individuals in receipt of antipsychotics), olanzapine (prescribed to 27%; 4269) and risperidone (22%; 3399) (online supplemental table 2). Similarly, among those without any record of SMI the three most commonly prescribed antipsychotics were: quetiapine (40%; 3657 of 9208 individuals in receipt of antipsychotics), olanzapine (21%; 1925) and risperidone (18%; 1660). Individuals may receive more than one type of antipsychotic agent so the percentages in online supplemental table 2 add up to more than 100%.

### Sensitivity analyses regarding the definition of adverse childhood experiences

We restricted the definition of adverse childhood experiences to a record occurring before age 17 years and a record of contact with social services. Of 1345 people who met this definition, 417 (31%) were prescribed antipsychotics. The rate ratios for all predictors of receiving antipsychotics were very similar to those when using the broader definition of adverse childhood experiences. People who met this definition had an aRR receiving antipsychotics of 1.45 (95% CI 1.22 to 1.71, p<0.001), compared with individuals without such a history in their health record.

In a second sensitivity analysis, we only included people with primary care data available from childhood (from at least age 11 years), to enable capture of adverse childhood experiences as recorded during childhood, rather than historically. There were 21 647 individuals with such data available (47% of our total PD cohort). Among these individuals, 1483 (7%) had records of social services contact from childhood, while 2805 (13%) had records in childhood referring to adverse childhood experiences (including but not limited to social services contact), and 3601 (17%) had records at any time referring to adverse childhood experiences (so including those with only retrospective records). The proportion of individuals within each of these groups prescribed antipsychotics were 34% (for childhood social service contact), 36% (record of trauma recorded in childhood) and 37% (record of childhood trauma recorded at any time). The aRR comparing those with and without social services contact in childhood, again among those without recorded SMI was 1.36 (95% CI 1.15 to 1.60, p<0.001). In summary, there was a statistically significant higher frequency of antipsychotic prescribing for people with a record of adverse childhood experiences history, which remained despite more rigorous definitions of childhood trauma.

## Discussion

### Principal findings

In this large UK-wide cohort of over 46 000 people with a record of PD diagnosis in their general practice notes, we found that more than one-third of people with PD were prescribed antipsychotics in UK primary care. Prescribing was common among people with PD even when there was no record of a diagnosis of SMI for which antipsychotics are usually indicated. These antipsychotic medications were prescribed for considerable lengths of time (frequently over a year) and prescribing has become more common over the period of our study to 2016. Among those with recorded PD but without recorded SMI, antipsychotic prescriptions were more common among women, individuals living in areas with greater deprivation, individuals with a record of EUPD or paranoid PD, and where the individual also had a history of adverse childhood experiences. The finding regarding adverse childhood experiences remained robust despite several analyses restricting the definition of adverse childhood experiences.

### Comparison with other studies

To our knowledge, this is the first study to investigate the pattern of antipsychotic prescribing among people with records of PD in UK primary care. Our findings suggest that while NICE guidance does not recommend long term prescribing of antipsychotics in treatment of PD, this practice is common in real life samples.^5^ There is some evidence that particular symptoms occurring within PD may be treated by antipsychotics: impulsivity, aggression, poor interpersonal relationships, global functioning and cognitive-perceptual symptoms are improved in meta-analyses of trials in EUPD.^22 23^ It is potentially understandable that clinicians would extrapolate these findings to the broader PD population in lieu of psychological treatments which may be harder to access for those with PD or may not be the patient’s preference for treatment. However, in our study, these antipsychotics are clearly being prescribed for considerable lengths of time and these treatments may not be reviewed or monitored appropriately, including for metabolic side effects.

We are not aware of any previous studies which examine antipsychotic medication according to history of adverse childhood experiences in people with PD. However, previous studies in SMI found that higher adverse childhood experience questionnaire scores are associated with higher antipsychotic prescribing, irrespective of diagnosis.^24^ Our findings are in line with this evidence from other diagnostic categories, but primary care data do not permit interrogation of the decision-making process underlying prescribing.

### Strengths and weaknesses

The strengths of our study include the large size of the PD cohort, enabling precise estimates and UK-wide coverage. While the THIN database pertains to approximately 6% of the UK population, previous studies have shown the THIN database to be socially and demographically representative of wider UK general practice,^13^ thus, the results may be cautiously generalised beyond the THIN database population to the wider UK general practice population. The study also benefits from complete records on the outcome (antipsychotics prescribed in primary care, including the type of antipsychotic), as all prescriptions are automatically computerised. Antipsychotics administered in secondary care, including some depot injections and clozapine would not be captured; the numbers in receipt of antipsychotics could be even higher if medication received in secondary care was also considered. We were not able to extract information on alternative treatment options, such as psychological therapy. All individuals with any record in their general practice notes of PD were included in the PD cohort. It is not possible to ascertain how the PD diagnoses were arrived at or to verify them. Therefore, there may be people in the PD cohort misclassified as having PD and some people with these problems will not be coded in the database. Similarly, only cases of SMI and adverse childhood experiences recorded using Read codes are captured. However, a previous study found prevalence of SMI diagnosis in THIN to be consistent with other epidemiological findings, suggesting that under recording is minimal.^19^ It is likely that some adverse childhood experiences will not be captured in the THIN data, where either the individual has not told their GP or the GP has not recorded this history as a Read code. The impact would be to underestimate the rate ratio for antipsychotic prescribing comparing those with and without adverse childhood experiences. However, sensitivity analyses restricting to a tighter definition of childhood contact with social services and limiting the PD cohort to those with records before aged 18 years and before aged 11 years did not meaningfully influence our main findings. A further limitation is that the clusters of PD severity used in other international settings are not coded in UK primary care. Results were not broken down by type of antipsychotic (first or second generation). However, since second generation antipsychotics are far more commonly prescribed in the cohort (online supplemental table), we would expect the patterns in prescribing of all antipsychotics to largely reflect patterns for second generation antipsychotics

### Interpretation, implications, conclusions and future research

We found that a considerable proportion of people with a record of PD in primary care had been prescribed antipsychotics, even when there was no recorded alternative indication (SMI record). A number of individuals received these medications for an extended period of time (at least 5 years). This is potentially concerning as longer term prescribing of antipsychotics is counter to current UK NICE guidelines, which do not support routine use of antipsychotics to treat PD (in the absence of SMI), on account of the limited evidence regarding their effectiveness for PD.^5^ The people in our PD cohort receiving antipsychotics will be at risk of experiencing adverse side effects from the drugs, possibly without any proven long term benefit of these agents for their mental health. They may develop adverse physical effects and yet may not receive appropriate physical health monitoring for weight gain and metabolic disturbance, because in the UK National Health Service physical health monitoring is currently only targeted at people with SMI. The lack of formal monitoring and protocols for prescribing antipsychotics in PD could also put people at risk of adverse effects of polypharmacy (eg, other psychotropic medications or medications for physical long-term conditions).

Possible explanations for the use of antipsychotics in PD could include lack of availability or long delays in access to other evidence based psychological therapies or because previous therapy/other treatments have not helped. We were unable to determine receipt of psychological therapies for emotional dysregulation and other problems as these therapies are usually provided in secondary care and not well recorded in primary care databases such as THIN. The use of antipsychotics could reflect patient or practitioner preference, due to belief in the effectiveness of antipsychotics for PD or indeed diagnostic uncertainty where mood instability or transient psychotic symptoms are present. It may be that the prescriptions issued in primary care are frequently provided as follow-on prescribing, after an initial prescription by a secondary care psychiatrist, without clear guidance on when to stop prescribing the antipsychotic drugs. The increase in antipsychotic prescriptions over time could reflect the impact of longer waiting lists for psychological interventions during a period of economic austerity, as well as general trends in prescribing of antipsychotics that has been observed for other conditions, and increased reporting of adverse childhood experiences, which were associated with higher levels of prescribing.^25^

There were variations in antipsychotic prescribing levels by sociodemographic characteristics. Notably, prescribing was higher in the more deprived areas, which could reflect poorer access to psychological therapies or higher levels of distress. Prescribing was also highest among those aged over 75 years, who would be particularly susceptible to the adverse metabolic effects of antipsychotics. Studies have previously shown antipsychotics being prescribed for dementia,^26^ despite also not recommended, which could explain some of the prescribing of antipsychotics in this age group.

The higher levels of prescribing in individuals with histories of adverse childhood experiences are not possible to explain fully with routine primary care data. People with records of adverse childhood experiences were more likely have a record of EUPD, compared with other PD types (almost half of all people with adverse childhood experiences, compared with one-quarter of those without), and were living in more deprived areas, both of these characteristics which were also associated with higher levels of antipsychotic prescriptions in our PD cohort. However, the increased rate of prescribing among people with adverse childhood experiences was observed even in multivariable models. This pattern is of particular concern as it is likely those with adverse childhood experiences might benefit more from appropriate psychological interventions as opposed to long-term medications.^27^

Further investigations are warranted to understand the reasons for longer term prescribing of antipsychotics in PD in primary care, particularly among those groups with higher prescribing levels. Future work should identify ways to address this and to promote recommended care pathways. Investigations of antipsychotic prescribing according to PD severity clusters is also warranted. Further research is needed to investigate the short and longer term effectiveness of antipsychotics in PD and the potential for adverse events in this group as well as the delivery of monitoring of such effects in primary care. This work would inform future guidelines regarding treatments for people with PD, to ultimately ensure people with PD receive the most appropriate, safe and effective treatment.

## Supplementary Material

Reviewer comments

Author's manuscript

## Data Availability

No data are available. No data are available as no new data collected. Read code list for personality disorder in online supplemental material.

## References

[1] Tyrer P, Reed GM, Crawford MJ. Classification, assessment, prevalence, and effect of personality disorder. Lancet 2015;385:717–26. 10.1016/S0140-6736(14)61995-4 25706217

[2] Samuels J. Personality disorders: epidemiology and public health issues. Int Rev Psychiatry 2011;23:223–33. 10.3109/09540261.2011.588200 21923224

[3] Moran P, Jenkins R, Tylee A, et al. The prevalence of personality disorder among UK primary care attenders. Acta Psychiatr Scand 2000;102:52–7. 10.1034/j.1600-0447.2000.102001052.x 10892610

[4] Bateman AW, Gunderson J, Mulder R. Treatment of personality disorder. Lancet 2015;385:735–43. 10.1016/S0140-6736(14)61394-5 25706219

[5] Kendall T, Pilling S, Tyrer P, et al. Borderline and antisocial personality disorders: summary of NICE guidance. BMJ 2009;338:b93. 10.1136/bmj.b93 19176682

[6] Gunderson JG. Clinical practice. Borderline personality disorder. N Engl J Med 2011;364:2037–42. 10.1056/NEJMcp1007358 21612472

[7] American Psychiatric Association Practice Guidelines. Practice guideline for the treatment of patients with borderline personality disorder. American psychiatric association. Am J Psychiatry 2001;158:1–52. 11665545

[8] Lieb K, Völlm B, Rücker G, et al. Pharmacotherapy for borderline personality disorder: cochrane systematic review of randomised trials. Br J Psychiatry 2010;196:4–12. 10.1192/bjp.bp.108.062984 20044651

[9] Paton C, Crawford MJ, Bhatti SF, et al. The use of psychotropic medication in patients with emotionally unstable personality disorder under the care of UK mental health services. J Clin Psychiatry 2015;76:e512–8. 10.4088/JCP.14m09228 25919844

[10] Newcomer JW. Antipsychotic medications: metabolic and cardiovascular risk. J Clin Psychiatry 2007;68 Suppl 4:8–13. 17539694

[11] Porter C, Palmier-Claus J, Branitsky A, et al. Childhood adversity and borderline personality disorder: a meta-analysis. Acta Psychiatr Scand 2020;141:6–20. 10.1111/acps.13118 31630389

[12] Cummins I. The impact of austerity on mental health service provision: a UK perspective. Int J Environ Res Public Health 2018;15:1145. 10.3390/ijerph15061145 PMC6025145 29865157

[13] Blak BT, Thompson M, Dattani H, et al. Generalisability of the health improvement network (thin) database: demographics, chronic disease prevalence and mortality rates. Inform Prim Care 2011;19:251–5. 10.14236/jhi.v19i4.820 22828580

[14] Chisholm J. The read clinical classification. BMJ 1990;300:1092. 10.1136/bmj.300.6732.1092 2344534 PMC1662793

[15] Davé S, Petersen I. Creating medical and drug code Lists to identify cases in primary care databases. Pharmacoepidemiol Drug Saf 2009;18:704–7. 10.1002/pds.1770 19455565

[16] Maguire A, Blak BT, Thompson M. The importance of defining periods of complete mortality reporting for research using automated data from primary care. Pharmacoepidemiol Drug Saf 2009;18:76–83. 10.1002/pds.1688 19065600

[17] Horsfall L, Walters K, Petersen I. Identifying periods of acceptable computer usage in primary care research databases. Pharmacoepidemiol Drug Saf 2013;22:64–9. 10.1002/pds.3368 23124958

[18] Townsend P. Deprivation. J Soc Policy 1987;16:125–46. 10.1017/S0047279400020341

[19] Hardoon S, Hayes JF, Blackburn R, et al. Recording of severe mental illness in United Kingdom primary care, 2000-2010. PLoS One 2013;8:e82365. 10.1371/journal.pone.0082365 24349267 PMC3861391

[20] Anda RF, Butchart A, Felitti VJ, et al. Building a framework for global surveillance of the public health implications of adverse childhood experiences. Am J Prev Med 2010;39:93–8. 10.1016/j.amepre.2010.03.015 20547282

[21] Woodman J, Freemantle N, Allister J, et al. Variation in recorded child maltreatment concerns in UK primary care records: a cohort study using the health improvement network (thin) database. PLoS One 2012;7:e49808. 10.1371/journal.pone.0049808 23209604 PMC3509120

[22] Nosè M, Cipriani A, Biancosino B, et al. Efficacy of pharmacotherapy against core traits of borderline personality disorder: meta-analysis of randomized controlled trials. Int Clin Psychopharmacol 2006;21:345–53. 10.1097/01.yic.0000224784.90911.66 17012981

[23] Vita A, De Peri L, Sacchetti E. Antipsychotics, antidepressants, anticonvulsants, and placebo on the symptom dimensions of borderline personality disorder: a meta-analysis of randomized controlled and open-label trials. J Clin Psychopharmacol 2011;31:613–24. 10.1097/JCP.0b013e31822c1636 21869691

[24] Anda RF, Brown DW, Felitti VJ, et al. Adverse childhood experiences and prescribed psychotropic medications in adults. Am J Prev Med 2007;32:389–94. 10.1016/j.amepre.2007.01.005 17478264 PMC3233770

[25] Marston L, Nazareth I, Petersen I, et al. Prescribing of antipsychotics in UK primary care: a cohort study. BMJ Open 2014;4:e006135. 10.1136/bmjopen-2014-006135 PMC4281533 25524544

[26] Martinez C, Jones RW, Rietbrock S. Trends in the prevalence of antipsychotic drug use among patients with Alzheimer’s disease and other dementias including those treated with antidementia drugs in the community in the UK: a cohort study. BMJ Open 2013;3:e002080. 10.1136/bmjopen-2012-002080 PMC3549220 23299113

[27] Nemeroff CB, Heim CM, Thase ME, et al. Differential responses to psychotherapy versus pharmacotherapy in patients with chronic forms of major depression and childhood trauma. Proc Natl Acad Sci U S A 2003;100:14293–6. 10.1073/pnas.2336126100 14615578 PMC283585

