# Supplementary material for: Prescribing of antipsychotics among people with recorded personality disorder in primary care: a retrospective nationwide cohort study using The Health Improvement Network primary care database
Source: BMJ Open. 2022 Mar 7;12(3):e053943. doi: 10.1136/bmjopen-2021-053943 (PMC8968526; doi:10.1136/bmjopen-2021-053943)
Supplement: Supplementary data [file bmjopen-2021-053943supp001.pdf]

Prescribing of antipsychotics among people with recorded Personality Disorder in primary care: a retrospective nationwide cohort study using The Health Improvement Network primary care database

Sarah L Hardoon[1], Joe F Hayes[1,2], Essi Viding[3], Eamon McCrory[3,4], Kate Walters[5], David PJ Osborn[1,2]

1 Division of Psychiatry, UCL, UK

2 Camden and Islington NHS Foundation Trust, UK

3 Division of Psychology and Language Sciences, UCL, UK

4 Anna Freud National Centre for Children and Families, UK

5 Department of Primary Care & Population Health, UCL, UK

Supplementary material

Appendix

Appendix Table 1 Read codes for personality disorder

Read code	Description	ICD-10 diagnostic category of PD	N*	%
E21..00	Personality disorders	other or non-specific	14321	30.99
E21y200	Borderline personality disorder	emotionally unstable	4926	10.66
E21z.00	Personality disorder NOS	other or non-specific	4676	10.12
E215200	Emotionally unstable personality	emotionally unstable	3477	7.52
E214100	Obsessional personality	anankastic	2564	5.55
Eu60300	[X]Emotionally unstable personality disorder	emotionally unstable	1988	4.30
E210.00	Paranoid personality disorder	paranoid	1760	3.81
E216.00	Inadequate personality disorder	dependent	1282	2.77
E212.00	Schizoid personality disorder	schizoid	998	2.16
E216.13	Labile personality	dependent	752	1.63
E21z.11	Psychopathic personality	dissocial	722	1.56
E21y500	Immature personality disorder	other or non-specific	605	1.31
E213.00	Explosive personality disorder	emotionally unstable	555	1.20
E215.11	Hysterical personality disorders	histrionic	531	1.15
E217.00	Antisocial or sociopathic personality disorder	dissocial	455	0.98
Eu60200	[X]Dissocial personality disorder	dissocial	411	0.89
Eu60z00	[X]Personality disorder, unspecified	other or non-specific	374	0.81
E215.00	Histrionic personality disorders	histrionic	369	0.80
E21y711	Neurotic personality	other or non-specific	357	0.77
E21yz00	Other personality disorder NOS	other or non-specific	352	0.76
Eu60600	[X]Anxious [avoidant] personality disorder	anxious	337	0.73
Eu60212	[X]Antisocial personality disorder	dissocial	326	0.71
Eu60512	[X]Obsessional personality disorder	anankastic	301	0.65
E214.00	Compulsive personality disorders	anankastic	249	0.54
Eu61.00	[X]Mixed and other personality disorders	other or non-specific	239	0.52
E216.12	Dependent personality	dependent	238	0.51
Eu60700	[X]Dependent personality disorder	dependent	229	0.50
E21y700	Psychoneurotic personality disorder	other or non-specific	210	0.45
Eu60513	[X]Obsessive-compulsive personality disorder	anankastic	206	0.45
Eu60311	[X]Aggressive personality disorder	emotionally unstable	205	0.44
E21y.00	Other personality disorders	other or non-specific	204	0.44
Eu60312	[X]Borderline personality disorder	emotionally unstable	176	0.38

Eu60214	[X]Psychopathic personality disorder	dissocial	158	0.34
E21y100	Avoidant personality disorder	anxious	145	0.31
E212z00	Schizoid personality disorder NOS	schizoid	126	0.27
Eu60411	[X]Hysterical personality disorder	histrionic	123	0.27
Eu60100	[X]Schizoid personality disorder	schizoid	122	0.26
E21y000	Narcissistic personality disorder	other or non-specific	111	0.24
Eu60000	[X]Paranoid personality disorder	paranoid	110	0.24
Eu60215	[X]Sociopathic personality disorder	dissocial	110	0.24
E214000	Anankastic personality	anankastic	106	0.23
E21..11	Neurotic personality disorder	other or non-specific	80	0.17
E21y400	Eccentric personality disorder	other or non-specific	64	0.14
Eu60500	[X]Anankastic personality disorder	anankastic	57	0.12
E214z00	Compulsive personality disorder NOS	anankastic	52	0.11
Eu60712	[X]Inadequate personality disorder	dependent	39	0.08
Eu60400	[X]Histrionic personality disorder	histrionic	37	0.08
E215z00	Histrionic personality disorder NOS	histrionic	35	0.08
Eu60y13	[X]Immature personality disorder	other or non-specific	35	0.08
Eu60014	[X]Sensitive paranoid personality disorder	paranoid	33	0.07
Eu60511	[X]Compulsive personality disorder	anankastic	27	0.06
Eu60.00	[X]Specific personality disorders	other or non-specific	27	0.06
E212000	Unspecified schizoid personality disorder	schizoid	26	0.06
Eu60z12	[X]Pathological personality NOS	other or non-specific	22	0.05
Eu60y00	[X]Other specific personality disorders	other or non-specific	21	0.05
E215000	Unspecified histrionic personality disorder	histrionic	20	0.04
E216.11	Asthenic personality	dependent	20	0.04
E21y300	Passive-aggressive personality disorder	other or non-specific	20	0.04
Eu60y16	[X]Psychoneurotic personality disorder	other or non-specific	17	0.04
E214.11	Anancastic personality	anankastic	11	0.02
Eu60713	[X]Passive personality disorder	dependent	11	0.02
Eu60313	[X]Explosive personality disorder	emotionally unstable	10	0.02
Eu60z11	[X]Character neurosis NOS	other or non-specific	10	0.02
Eu60711	[X]Asthenic personality disorder	dependent	9	0.02
Eu60y14	[X]Narcissistic personality disorder	other or non-specific	8	0.02
Eu60y11	[X]Eccentric personality disorder	other or non-specific	6	0.01
Eu60714	[X]Self defeating personality disorder	dependent	4	0.01
Eu60213	[X]Asocial personality disorder	dissocial	3	0.01
E21y600	Masochistic personality disorder	other or non-specific	3	0.01
E217.11	Amoral personality	dissocial	2	<0.01
E215300	Psychoinfantile personality	histrionic	1	<0.01
Eu60412	[X]Psychoinfantile personality disorder	histrionic	1	<0.01
Eu60y12	[X]Haltlose type personality disorder	other or non-specific	1	<0.01
E210.11	Fanatic personality	paranoid	0	0
Eu60011	[X]Expansive paranoid personality disorder	paranoid	0	0
Eu60012	[X]Fanatic paranoid personality disorder	paranoid	0	0
Eu60013	[X]Querulant personality disorder	paranoid	0	0
Eu60211	[X]Amoral personality disorder	dissocial	0	0
Eu60y15	[X]Passive-aggressive personality disorder	other or non-specific	0	0

*N = number in cohort with the Read code as their most recent specific Read code for PD, unless individual had only non-specific Read codes, in which case, instead it is the most recent non-specific Read code.

Appendix Table 2 Types of antipsychotics prescribed

	Typical (T) or Atypical (A)	All prescribed antipsychotics, n = 15562		Prescribed antipsychotics, SMI, n = 6354		All prescribed antipsychotics, no SMI, n = 9208	
		N	(%)	N	(%)	N	(%)
Atypical		12007	(77.2)	5370	(84.5)	6637	(72.1)
Typical		6688	(43.0)	2756	(43.4)	3932	(42.7)
QUETIAPINE	A	5819	(37.4)	2162	(34.0)	3657	(39.7)
OLANZAPINE	A	4269	(27.4)	2344	(36.9)	1925	(20.9)
RISPERIDONE	A	3399	(21.8)	1739	(27.4)	1660	(18.0)
CHLORPROMAZINE	T	2426	(15.6)	968	(15.2)	1458	(15.8)
ARIPIPRAZOLE	A	1728	(11.1)	1015	(16.0)	713	(7.7)
HALOPERIDOL	T	1298	(8.3)	606	(9.5)	692	(7.5)
FLUPENTIXOL	T	1041	(6.7)	459	(7.2)	582	(6.3)
TRIFLUOPERAZINE	T	951	(6.1)	407	(6.4)	544	(5.9)
AMISULPRIDE	A	854	(5.5)	549	(8.6)	305	(3.3)
PROMAZINE	T	666	(4.3)	215	(3.4)	451	(4.9)
THIORIDAZINE	T	560	(3.6)	212	(3.3)	348	(3.8)
SULPIRIDE	T	472	(3.0)	303	(4.8)	169	(1.8)
ZUCLOPENTHIXOL	T	365	(2.3)	256	(4.0)	109	(1.2)
LEVOMEPROMAZINE	T	354	(2.3)	90	(1.4)	264	(2.9)
PERICYAZINE	T	245	(1.6)	77	(1.2)	168	(1.8)
CLOZAPINE	A	146	(0.9)	129	(2.0)	17	(0.2)
FLUPHENAZINE	T	122	(0.8)	77	(1.2)	45	(0.5)
PERPHENAZINE	T	65	(0.4)	19	(0.3)	46	(0.5)
PIPOTIAZINE	T	43	(0.3)	36	(0.6)	7	(0.1)
PALIPERIDONE	T	41	(0.3)	33	(0.5)	8	(0.1)
DROPERIDOL	T	23	(0.1)	18	(0.3)	5	(0.1)
PIMOZIDE	T	19	(0.1)	15	(0.2)	4	(0.1)
BENPERIDOL	T	14	(0.1)	7	(0.1)	7	(0.1)
LURASIDONE	A	7	(0.1)	6	(0.1)	1	(0.1)
ASENAPINE	A	5	(0.1)	3	(0.1)	2	(0.1)
ZOTEPINE	A	5	(0.1)	4	(0.1)	1	(0.1)
LOXAPINE	T	2	(0.1)	1	(0.1)	1	(0.1)
SERTINDOLE	A	2	(0.1)	2	(0.1)	0	(0.1)
OXYPERTINE	T	1	(0.1)	1	(0.1)	0	(0.1)

Individuals may receive more than one type of antipsychotic so percentages add up to more than 100%. Other antipsychotics considered but which were not prescribed to anyone in the cohort: Chlorprothixene, Fluspirilene, Remoxipride, Thiopropazate, Thioproperazine, Trifluperidol.

Appendix Table 3 Proportions of cohort with repeat prescriptions and at least 6 months, 1 year, 3 years and 5 years continuous antipsychotic use

	Total present for time period	With length of antipsychotic use, n (%)		Total with recorded SMI present for time period	With length of antipsychotic use, n (%)		Total with no recorded SMI present for time period	With length of antipsychotic use, n (%)	
**At least two prescriptions ≤ 84 days apart**	46210	13898	(30.1)	9335	5973	(64.0)	36875	7925	(21.5)
**≥6 months continuous use**	40850	11906	(29.1)	8281	5358	(64.7)	32569	6548	(20.1)
**≥1 year continuous use**	35794	9588	(26.8)	7218	4591	(63.6)	28576	4997	(17.5)
**≥3 year continuous use**	24081	5492	(22.8)	4741	2954	(62.3)	19340	2538	(13.1)
**≥5 year continuous use**	17588	3529	(20.1)	3385	2044	(60.4)	14203	1485	(10.5)
