# Supplementary material for: Prescribing of antipsychotics among people with recorded personality disorder in primary care: a retrospective nationwide cohort study using The Health Improvement Network primary care database
Source: BMJ Open. 2022 Mar 7;12(3):e053943. doi: 10.1136/bmjopen-2021-053943 (PMC8968526; doi:10.1136/bmjopen-2021-053943)
Supplement: Author's manuscript [file bmjopen-2021-053943.draft_revisions.pdf]

BMJ Open is committed to open peer review. As part of this commitment we make the peer review history of every article we publish publicly available.

When an article is published we post the peer reviewers' comments and the authors' responses online. We also post the versions of the paper that were used during peer review. These are the versions that the peer review comments apply to.

The versions of the paper that follow are the versions that were submitted during the peer review process. They are not the versions of record or the final published versions. They should not be cited or distributed as the published version of this manuscript.

BMJ Open is an open access journal and the full, final, typeset and author-corrected version of record of the manuscript is available on our site with no access controls, subscription charges or pay-per-view fees (http://bmjopen.bmj.com).

If you have any questions on BMJ Open's open peer review process please email
info.bmjopen@bmj.com

BMJ Open

Prescribing of antipsychotics among people with recorded Personality Disorder in primary care: a retrospective nationwide cohort study using The Health Improvement Network primary care database

Journal:	*BMJ Open*
Manuscript ID	bmjopen-2021-053943
Article Type:	Original research
Date Submitted by the Author:	29-May-2021
Complete List of Authors:	Hardoon, Sarah; UCL, Division of Psychiatry<br>Hayes, Joseph; UCL<br>Viding, Essi; UCL<br>McCrory, Eamon; UCL<br>Walters, Kate; UCL<br>Osborn, David; UCL
Keywords:	Personality disorders < PSYCHIATRY, PRIMARY CARE, THERAPEUTICS

SCHOLARONE™
Manuscripts

I, the Submitting Author has the right to grant and does grant on behalf of all authors of the Work (as defined in the below author licence), an exclusive licence and/or a non-exclusive licence for contributions from authors who are: i) UK Crown employees; ii) where BMJ has agreed a CC-BY licence shall apply, and/or iii) in accordance with the terms applicable for US Federal Government officers or employees acting as part of their official duties; on a worldwide, perpetual, irrevocable, royalty-free basis to BMJ Publishing Group Ltd ("BMJ") its licensees and where the relevant Journal is co-owned by BMJ to the co-owners of the Journal, to publish the Work in this journal and any other BMJ products and to exploit all rights, as set out in our licence.

The Submitting Author accepts and understands that any supply made under these terms is made by BMJ to the Submitting Author unless you are acting as an employee on behalf of your employer or a postgraduate student of an affiliated institution which is paying any applicable article publishing charge ("APC") for Open Access articles. Where the Submitting Author wishes to make the Work available on an Open Access basis (and intends to pay the relevant APC), the terms of reuse of such Open Access shall be governed by a Creative Commons licence – details of these licences and which Creative Commons licence will apply to this Work are set out in our licence referred to above.

Other than as permitted in any relevant BMJ Author's Self Archiving Policies, I confirm this Work has not been accepted for publication elsewhere, is not being considered for publication elsewhere and does not duplicate material already published. I confirm all authors consent to publication of this Work and authorise the granting of this licence.

For peer review only

Prescribing of antipsychotics among people with recorded Personality Disorder in primary care: a retrospective nationwide cohort study using The Health Improvement Network primary care database

Sarah L Hardoon[1], Joe F Hayes[1,2], Essi Viding[3], Eamon McCrory[3,4], Kate Walters[5], David PJ Osborn[1,2*]

1 Division of Psychiatry, UCL, UK

2 Camden and Islington NHS Foundation Trust, UK

3 Division of Psychology and Language Sciences, UCL, UK

4 Anna Freud National Centre for Children and Families, UK

5 Department of Primary Care & Population Health, UCL, UK

*Corresponding author; email: d.osborn@ucl.ac.uk

Abstract

Objectives Investigate the frequency of antipsychotic prescribing to people with recorded personality disorder (PD) in United Kingdom (UK) primary care and factors associated with such prescribing.

Design Retrospective cohort study

Setting General practices contributing to The Health Improvement Network UK-wide primary care database, 1 January 2000 – 31 December 2017

Participants 46,210 people registered with participating general practices who had a record of PD in their general practice notes

Main outcome measures Prescriptions for antipsychotics in general practice records and length of time in receipt of antipsychotic prescriptions

Results Of 46,210 people with recorded PD, 15,562 (34%) were ever prescribed antipsychotics. Among the subgroup of 36,875 people with recorded PD, but no recorded severe mental illness (SMI), 9,208 (25%) were prescribed antipsychotics; prescribing increased with deprivation (adjusted rate ratio (aRR) comparing least to most deprived quintile: 0.56, 95% CI 0.48-0.66, p<0.001), was higher in females (aRR:1.25, 95% CI 1.16-1.34, p<0.001), and with a history of adverse childhood experiences (aRR:1.44, 95% CI 1.28-1.56, p<0.001). Median time prescribed antipsychotics was 605 days (IQR 197-1639 days). Prescribing frequency has increased over time.

Conclusions Contrary to current UK guidelines, antipsychotics are frequently and increasingly prescribed to people with recorded PD, for extended periods. An urgent review of clinical practice is warranted, including the effectiveness of such prescribing and the need to monitor for adverse effects, including metabolic complications.

For peer review only - http://bmjopen.bmj.com/site/about/guidelines.xhtml

Article summary: Strengths and limitations of this study

- In the present report we identified a very large UK-wide cohort of >46,000 people with a recorded personality disorder diagnosis, enabling, for the first time, a detailed UK-wide exploration of prescribing practices in personality disorder (PD) over an 18-year period from 2000 to 2017.
- The cohort was derived from The Health Improvement Network primary care database which has been shown to be socially and demographically representative of wider UK general practice.
- The study benefits from complete records on the outcome (antipsychotics prescribed in primary care, including the type of antipsychotic), as all prescriptions are automatically computerised.
- Potential limitations include limited information on alternative treatment options, such as psychological therapy, and that it is not possible to ascertain how the PD diagnoses were arrived at or to verify them.

For peer review only

Introduction

The core features of personality disorder (PD) diagnoses are pervasive patterns of behaviour and maladaptive traits leading to substantial personal distress, social dysfunction and disruption to others, often beginning in early adult life(1). Prevalence estimates for PD vary globally and by clinical setting(2), with limited research regarding the prevalence in primary care settings(3). There is limited evidence on the effectiveness of antipsychotic pharmacotherapy in PD. Results from randomised controlled trials mainly focus on EUPD populations, but the findings are limited by short term follow-up, small sample sizes and variability in outcome measures (4). In the UK, treatment guidelines (from the National Institute for Health and Care Excellence (NICE)) exist for EUPD and dissocial PD. For both diagnoses it is recommended that “drug treatment should not be used”, apart from short-term sedating medication during crises in EUPD(5). This is echoed by guidelines for EUPD from Australia’s National Health and Medical Research Council(6). Conversely, the American Psychiatric Association recommends targeted pharmacotherapy for PD: antidepressant medication for affective instability, mood stabilisers for impulsive aggression and antipsychotics for cognitive-perceptive disturbance(7). Cochrane reviews find no evidence for the efficacy of antidepressant medication, but some evidence that mood stabilisers may reduce affective dysregulation and impulsive–aggression and that antipsychotic medication may improve cognitive–perceptual symptoms and affective dysregulation in PD(8). Despite this mixed evidence, and counter to the NICE guidelines, there is some limited data suggesting that psychotropic prescribing for PD is occurring in the UK mental health service setting(9). There is an urgent need to ascertain the wider extent and duration of psychotropic prescribing in PD. There is a particular concern regarding the prescribing of antipsychotics given their adverse event profile, including metabolic and cardiovascular risks(10). Further, the characteristics of individuals prescribed psychotropic medications need to be determined to understand who may be most likely to receive these medications. For example, females are more likely than males to be diagnosed with borderline or EUPD and there is a high prevalence of adverse childhood experiences among those with EUPD(11).

For peer review only - http://bmjopen.bmj.com/site/about/guidelines.xhtml

Yet it is not known whether patients of particular gender, PD diagnosis or life history are more likely to receive antipsychotic medication. Time trends in antipsychotic prescribing for PD have not been examined and may be important, as UK mental health services and social care provision have been negatively affected by austerity(12). This study aimed to determine how frequently antipsychotics are prescribed for people with a diagnosis of PD in UK primary care as well as factors associated with this prescribing. The three main aims of the study were: 1) To quantify the prevalence and duration of antipsychotic prescribing among patients with a record of PD, particularly those without any diagnostic record of SMI; 2) To describe time trends in prescribing of antipsychotics 3) To determine whether gender, age, area level deprivation, mental health comorbidities, category of PD diagnosis or a recorded history of adverse childhood experiences affect the likelihood of being prescribed antipsychotics; 3) To examine time trends in prescribing of antipsychotics. We hypothesised that people experiencing greater levels of deprivation and with adverse childhood experiences might be more likely to receive antipsychotic treatment.

Methods

Study design

Retrospective nationwide cohort study

Data source

We used data from The Health Improvement Network (THIN) primary care database, which comprised, at time of analysis, computerised anonymised patient records retrieved from 744 general practices across the UK(13).

The database includes all diagnoses, symptoms and other health data (blood tests, health indicators) recorded by the general practitioner (GP) in the patient's computerised records, as well as all prescriptions issued. Diagnoses, symptoms and other health information in THIN are coded using the Read code clinical classification system(14). The database has been shown to be broadly representative of the UK general population(13).

Study population

From the THIN database population we identified the smaller study population comprising people registered with a participating THIN general practice for at least some part of the period 2000-2017, who had a record in their general practice notes at any time during or prior to this period of a diagnosis of PD (specific or non-specific), identified via the presence of a Read code indicative of a PD diagnosis. A list of relevant Read codes was developed using established search techniques(15) and with reference to ICD-10 chapters F60-F61. The diagnostic list was verified by a clinician (JFH). The complete Read code list is provided in the Appendix (Supplementary material, Appendix Table 1). All permanently registered patients were included from the date the general practice reached agreed data quality thresholds: acceptable mortality rate date(16) and acceptable computer usage date(17).

PD Read codes were categorised in line with current versions of the International Classification of Diseases (ICD-10) and the Diagnostic and Statistical Manual of Mental Disorders (DSM-5) as: paranoid, schizoid, dissocial, EUPD (including borderline), histrionic, anankastic, anxious/avoidant, dependent and other (chiefly comprising codes for non-specific PD, but also including diagnoses such as eccentric, haltlose, immature, narcissistic, masochistic, passive-aggressive and mixed). Where an

For peer review only - http://bmjopen.bmj.com/site/about/guidelines.xhtml

individual had more than one type of specific PD record, the most recent record was assigned as their diagnosis.

Outcomes

The primary outcome was a prescription for antipsychotic medication in the individual's primary care records, any time after the initial PD diagnosis. We included all first generation and second generation antipsychotics in British National Formulary (BNF) chapters 4.2.1; 4.2.2 (listed in Supplementary material, Appendix Table 2).

It is not possible to ascertain from THIN records whether a prescription that was issued was actually dispensed or used by the individual. Therefore we also included an outcome of at least two consecutive prescriptions being issued fewer than 84 days apart as a likely indication that the first prescription had been used (corresponds to 3 x 28 days, a common period for which longer term medicines are issued in UK primary care).

We also determined the length in days of continuous antipsychotic treatment. A continuous treatment period was defined as repeat prescriptions for antipsychotics no more than 84 days apart. The length of a continuous treatment period was then calculated from the date of the first prescription in the period to date of last prescription + 84 days.

Follow-up period

Cohort entry was defined as the latest date of: registration at the general practice, date practice reached data quality thresholds, date of first PD diagnosis record, or 1 January 2000. End of follow-up for an individual was the earliest of: date of death, date patient transferred out of practice, latest date practice contributed data to THIN, or 31 December 2016.

Covariates

Antipsychotic prescribing was explored according to the following sociodemographic characteristics: gender, age and area level social deprivation (using the Townsend score which is based on individual's postcode and grouped into quintiles(18)).

We extracted data regarding comorbid mental health conditions defined as: severe mental illness (SMI, including schizophrenia, bipolar disorder, and other non-organic psychotic illness), depression, anxiety, phobia, hypochondria, obsessive compulsive disorder, other neurosis, eating disorder, post-traumatic stress disorder. Each of these conditions were identified by the presence of a Read code at any time, using lists of Read codes developed through established techniques, with reference to relevant ICD chapters and verified by clinician (JFH).

The presence of recorded adverse childhood experiences was identified from records with indicative Read codes, including both codes recorded in childhood (when individual <17 years) relating to contact with social services, or referring directly to physical or emotional abuse, or codes recorded during adulthood indicative of a history of childhood abuse, or history of contact with social services as a child. Again, established search techniques were used to develop the Read code list, updating previous work on childhood maltreatment(19), extending the code list from 500+ to 1000+ codes, reflecting the addition of new codes to the Read code dictionary.

For peer review only - http://bmjopen.bmj.com/site/about/guidelines.xhtml

Patient and public involvement

Service users were involved in the study through participation in the Pathfinder Data Science PPI Group, with the McPin Foundation. The group were involved in the design, conduct and reporting plans for our research.

Statistical analysis

We determined the prevalence of the following outcomes: i) receiving at least one antipsychotic prescription, ii) at least two antipsychotic prescriptions fewer than 84 days apart as well as for continuous treatment periods of at least iii) six months, iv) one year, v) three years, vi) five years. Prevalence estimates were computed for the whole sample and then by: gender, age (in 10 year age bands), Townsend deprivation quintile, diagnostic category of PD, recorded mental health comorbidity, and whether an individual had a recorded history of adverse childhood experiences. Analyses were stratified by whether patients ever had an additional recorded diagnosis of SMI. For the prevalence estimates of continuous treatment periods the denominators were restricted to individuals who had the relevant period of follow-up in the database in order to be 'at risk'.

Rate ratios for antipsychotic prescribing were estimated from univariable and multivariable Poisson regression, again stratified by ever receiving a diagnosis of SMI. Multilevel Poisson models were used, with individuals nested in general practices, to account for potential clustering of individuals in general practices and clustering of prescribing practices within general practices.

Time trends in antipsychotic prescribing were determined as yearly prevalence estimates of antipsychotic prescriptions: namely the number within the sample with at least one antipsychotic prescription in that year/ number of the sample present in the database that year.

We calculated the median and interquartile range of length of longest continuous treatment period for all antipsychotics and for individual agents. The numbers of individuals receiving at least one prescription for individual agents were also computed.

We carried out sensitivity analyses using a stricter definition of adverse childhood experiences of those with a record before age 17 years specifically referring to contact with social services, and restricting the cohort to those individuals with records from at least age 11 years, thus with records from childhood.

Results

Our cohort comprised 46,210 people who had a record of PD in their general practice notes. Median duration in the study was 3.2 years (IQR 1.1 to 8.0 years). Just over half the cohort were female (23,528, 51%), and the average age at study entry was 43 years (Table 1). The cohort had greater levels of deprivation relative to the general population, with 26% of people living in areas within the most deprived quintile, and 12% in the least deprived quintile. The most commonly recorded diagnostic category of PD was EUPD recorded among 12,300 people (27% of the cohort), followed by anankastic (3,648, 8%). However, 44% of the cohort had only non-specific PD records including primarily 14321 (31%) with Read code E21..00 "Personality Disorders" and 4676 (10%) with Read code E21z.00 "Personality Disorders NOS" (Supplementary material, Appendix Table 1). A record of ever being diagnosed with an SMI was present in 9,335 people (20% of the PD cohort), while 34,522 (75%) and 23,595 (51%) had records of depression and anxiety respectively. A history of adverse childhood experiences was recorded for 4,742 people (10%) in the cohort.

For peer review only - http://bmjopen.bmj.com/site/about/guidelines.xhtml

Prevalence of antipsychotic prescribing

One third of the cohort of people with recorded diagnosis of PD were prescribed antipsychotics at least once after their first PD record (15,562, 34%, Table 2). The majority of those prescribed antipsychotics (9,208, 59%) in the cohort had no record of SMI to explain the prescribing. Indeed, restricting the cohort to 36,875 individuals with recorded PD but no record of SMI, one quarter (9,208, 25%) had been prescribed antipsychotics

Patterns of prescribing by gender, age, deprivation, mental health comorbidity, type of PD and recorded history of adverse childhood experiences, are shown in Table 2. Among those with recorded PD but *without* recorded SMI, prescribing of antipsychotics was higher among females than males (28% versus 22%) and highest among those aged 20-34 years and over 75 years, compared to other age groups. Prescribing increased with deprivation, from 19% in the least deprived quintile to 29% in the most deprived quintile. There was some variation in prescribing according to diagnostic category of PD, with prescribing highest among those with record of EUPD (38%), followed by paranoid PD (33%). Those who had a recorded history of adverse childhood experiences in their GP record were more likely to be prescribed antipsychotics than those who did not (31% versus 24%).

Adjusted and unadjusted rate ratios comparing rates of prescribing of antipsychotics for different levels of the socio-demographic characteristics, are shown in Table 3. The differences in prescribing of antipsychotics by gender, age, and deprivation, type of PD and record of adverse childhood experience all remained significant after adjustment in the group with no additional record of SMI.

Time trends

The frequency of prescribing of antipsychotics increased over time: Of those individuals who were present in the cohort in 2000, 17% received antipsychotics. Of those individuals present in the cohort in 2016, 26% received antipsychotics (Fig 1). Among those without a record of SMI, prescribing increased from 9% in 2000 to 19% in 2016.

Duration of antipsychotic treatment in personality disorder

The median duration of continuous prescriptions of antipsychotics was 605 days (IQR 197 to 1639 days). Among those with no recorded SMI, the median duration of continuous treatment was 439 days (IQR 150 to 1218 days). For each of these results we only assessed the longest period of treatment for each individual.

Among the total cohort of 46,210 people, 30% received at least two consecutive antipsychotic prescriptions within 84 days, and substantial numbers were prescribed antipsychotics over at least six months (29%), one year (27%), three years (23%) and five years (20%) (Supplementary material, Appendix table 3). Among the subgroup of individuals without recorded SMI, 22% received at least two consecutive prescriptions within 84 days, and many were prescribed antipsychotics for more than six months (20%), one year (18%), three years (13%) and five years (11%).

Individual antipsychotics agents

The most commonly prescribed antipsychotics in the cohort were Quetiapine (prescribed to 37% of all those in receipt of antipsychotics), Olanzapine (prescribed to 27%) and Risperidone (22%) (Supplementary material, Appendix table 2). Similarly, among those without any record of SMI the

For peer review only - http://bmjopen.bmj.com/site/about/guidelines.xhtml

three most commonly prescribed antipsychotics were: Quetiapine (40%), Olanzapine (21%) and Risperidone (18%).

Sensitivity analyses regarding the definition of adverse childhood experiences

We restricted the definition of adverse childhood experiences to a record occurring before age 17 years and a record of contact with social services. Of 1,345 people who met this definition, 417 (31%) were prescribed antipsychotics. The rate ratios for all predictors of receiving antipsychotics were very similar to those when using the broader definition of adverse childhood experiences. People who met this definition has an adjusted rate ratio for receiving antipsychotics of 1.45 (95% CI 1.22 to 1.71, p<0.001), compared to individuals without such a history in their health record.

In a second sensitivity analysis, we only included people with primary care data available from childhood (from at least age 11 years), to enable capture of adverse childhood experiences as recorded during childhood, rather than historically. There were 21,647 individuals with such data available (47% of our total cohort). Among these individuals 1,483 (7%) had records of social services contact from childhood, while 2,805 (13%) had records in childhood referring to adverse childhood experiences (including but not limited to social services contact), and 3,601 (17%) had records at any time referring to adverse childhood experiences (so including those with only retrospective records). The proportion of individuals within each of these groups prescribed antipsychotics were 34% (for childhood social service contact), 36% (record of trauma recorded in childhood) and 37% (record of childhood trauma recorded at any time). The adjusted rate ratio comparing those with and without social services contact in childhood, again among those without recorded SMI was 1.36 (95% CI 1.15 to 1.60, p<0.001). In summary, there was a statistically significant higher frequency of antipsychotic prescribing for people with a record of adverse childhood experiences history, which remained despite more rigorous definitions of childhood trauma.

Discussion

Principal findings

In this large UK-wide cohort of over 46,000 people with a record of PD diagnosis in their general practice notes, we found that more than a third of people with PD are prescribed antipsychotics in UK primary care. Prescribing was common amongst people with PD even when there was no record of a diagnosis of SMI for which antipsychotics are usually indicated. These antipsychotic medications were prescribed for considerable lengths of time (frequently over a year) and prescribing has become more common over the period of our study to 2017. Among those with recorded PD but *without* recorded SMI, antipsychotic prescriptions were more common among women, individuals with greater area level deprivation, individuals with a record of EUPD or paranoid PD, and where the individual also had a history of adverse childhood experiences. The finding regarding adverse childhood experiences remained robust despite several analyses restricting the definition of adverse childhood experiences.

Comparison with other studies

To our knowledge this is the first study to investigate the pattern of antipsychotic prescribing among people with records of PD in UK primary care. Our findings suggest that while NICE guidance does

For peer review only - http://bmjopen.bmj.com/site/about/guidelines.xhtml

not recommend long term prescribing of antipsychotics in treatment of PD, this practice is common in real life samples (5). There is some evidence that particular symptoms occurring within PD may be treated by antipsychotics: impulsivity, aggression, poor interpersonal relationships, global functioning and cognitive-perceptual symptoms are improved in meta-analyses of trials in EUPD(20;21). It is potentially understandable that clinicians would extrapolate these findings to the broader PD population in lieu of psychological treatments which may be harder to access for those with PD or may not be the patient's preference for treatment. However, in our study these antipsychotics are clearly being prescribed for considerable lengths of time and these treatments may not reviewed or monitored appropriately, including for metabolic side effects.

We are not aware of any previous studies which examine antipsychotic medication according to history of adverse childhood experiences in people with PD. However, previous studies in SMI found that higher adverse childhood experience questionnaire scores are associated with higher antipsychotic prescribing, irrespective of diagnosis(22). Our findings are in line with this evidence from other diagnostic categories, but primary care data do not permit interrogation of the decision-making process underlying prescribing.

Strengths and weaknesses

The strengths of our study include the large size of the cohort, enabling precise estimates, and UK-wide coverage. Previous studies have shown the THIN database to be socially and demographically representative of wider UK general practice(13), thus the results may be generalised beyond the THIN database to the wider UK population. The study also benefits from complete records on the outcome (antipsychotics prescribed in primary care, including the type of antipsychotic), as all prescriptions are automatically computerised. Antipsychotics administered in secondary care, including some depot injections and clozapine would not be captured; the numbers in receipt of antipsychotics could be even higher if medication received in secondary care was also considered. We were not able to extract information on alternative treatment options, such as psychological therapy. All individuals with any record in their general practice notes of PD were included in the cohort. It is not possible to ascertain how the PD diagnoses were arrived at or to verify them. Therefore, there may be people in the cohort misclassified as having PD and some people with these problems will not be coded in the database. Similarly, only cases of SMI and adverse childhood experiences recorded using Read codes are captured. However, a previous study found prevalence of SMI diagnosis in THIN to be consistent with other epidemiological findings, suggesting that under recording is minimal(23). It is likely that some adverse childhood experiences will not be captured in the THIN data, where either the individual has not told their GP or the GP has not recorded this history as a Read code. The impact would be to underestimate the rate ratio for antipsychotic prescribing comparing those with and without adverse childhood experiences. However, sensitivity analyses restricting to a tighter definition of childhood contact with social services and limiting the cohort to those with records before aged 18 and before aged 11 did not meaningfully influence our main findings.

Interpretation, implications, conclusions and future research

We found that a considerable proportion of people with a record of PD in primary care had been prescribed antipsychotics, even when there was no recorded alternative indication (SMI record). A number of individuals received these medications for an extended period of time (at least 5 years). This is potentially concerning as longer term prescribing of antipsychotics is counter to current UK NICE guidelines, which do not support routine use of antipsychotics to treat PD (in the absence of SMI), on account of the limited evidence regarding their effectiveness for PD(5). The people in our

For peer review only - http://bmjopen.bmj.com/site/about/guidelines.xhtml
For peer review only

cohort receiving antipsychotics will be at risk of experiencing adverse side-effects from the drugs, possibly without any proven long term benefit of these agents for their mental health. They may develop adverse physical effects and yet may not receive appropriate physical health monitoring for weight gain and metabolic disturbance, because in the UK NHS physical health monitoring is currently only targeted at people with SMI.

Possible explanations for the use of antipsychotics in PD could include lack of availability or long delays in access to other evidence based psychological therapies or because previous therapy/ other treatments have not helped. We were unable to determine receipt of psychological therapies for emotional dysregulation and other problems as these therapies are usually provided in secondary care and not well recorded in primary care databases such as THIN. The use of antipsychotics could reflect patient or practitioner preference, due to belief in the effectiveness of antipsychotics for PD or indeed diagnostic uncertainty where mood instability or transient psychotic symptoms are present. It may be that the prescriptions issued in primary care are frequently provided as follow-on prescribing, after an initial prescription by a secondary care psychiatrist, without clear guidance on when to stop prescribing the antipsychotic drugs. The increase in antipsychotic prescriptions over time could reflect the impact of longer waiting lists for psychological interventions during a period of austerity, as well as general trends in prescribing of antipsychotics that has been observed for other conditions(24).

There were variations in antipsychotic prescribing levels by socio-demographic characteristics. Notably, prescribing was higher in the more deprived areas, which could reflect poorer access to psychological therapies or higher levels of distress. Prescribing was also highest among those aged over 75 years, who would be particularly susceptible to the adverse metabolic effects of. Studies have previously shown antipsychotics being prescribed for dementia(25), despite also not recommended, which could explain some of the prescribing of antipsychotics in this age group.

The higher levels of prescribing in individuals with histories of adverse childhood experiences are not possible to explain fully with routine primary care data. People with records of adverse childhood experiences were more likely have a record of EUPD, compared with other PD types (almost half of all people with adverse childhood experiences, compared with one quarter of those without), and were living in more deprived areas, both of these characteristics which were also associated with higher levels of antipsychotic prescriptions in our cohort. However, the increased rate of prescribing among people with adverse childhood experiences was observed even in multivariable models. This pattern is of particular concern as it is likely those with adverse childhood experiences might benefit more from appropriate psychological interventions as opposed to long term medications(26).

Further investigations are warranted to understand the reasons for longer term prescribing of antipsychotics in PD in primary care, particularly among those groups with higher prescribing levels. Future work should identify ways to address this and to promote recommended care pathways. Further research is also needed to investigate the short and longer term effectiveness of antipsychotics in PD and the potential for adverse events in this group as well as the delivery of monitoring of such effects in primary care. This work would inform future guidelines regarding treatments for people with PD, to ultimately ensure people with PD receive the most appropriate, safe and effective treatment.

For peer review only - http://bmjopen.bmj.com/site/about/guidelines.xhtml

Ethics statement: Anonymised data were used throughout the study provided by the data provider to UCL. Studies using The Health Improvement Network (THIN) database have had initial ethical approval from the NHS South-East Multicentre Research Ethics Committee, subject to prior independent scientific review. The Scientific Review Committee (IQVIA) approved the study protocol (SRC Reference Number: 17THIN072) prior to its undertaking.

Competing interests: None declared

Funding Statement: This work was supported by the Medical Research Council (grant number MC_PC_17216); and the Wellcome Trust (JFH, grant number 211085/Z/18/Z)

Acknowledgments: The Pathfinder Data Science PPI Group, led by Dan Robotham, from the McPin Foundation for their input and feedback on the study. DPJO and JFH are supported by the University College London Hospitals NIHR Biomedical Research Centre and the NIHR North Thames Applied Research Collaboration. This funder had no role in study design, data collection, data analysis, data interpretation, or writing of the report. The views expressed in this article are those of the authors and not necessarily those of the NHS, the NIHR, or the Department of Health and Social Care.

Author contribution: SLH: formulation of research questions, study design, extraction and analysis of data, interpretation of data, writing of first draft of paper, critical revision of paper for important intellectual content, approval of final draft. JFH: formulation of research questions, study design, acquisition of data, interpretation of data, writing of first draft of paper, critical revision of paper for important intellectual content, approval of final draft. EV: formulation of research questions, study design, interpretation of data, critical revision of paper for important intellectual content, approval of final draft. EM: formulation of research questions, study design, interpretation of data, critical revision of paper for important intellectual content, approval of final draft. KW: interpretation of data, critical revision of paper for important intellectual content, approval of final draft. DPJO: formulation of research questions, study design, interpretation of data, critical revision of paper for important intellectual content, approval of final draft.

Data sharing statement: No data are available as no new data collected. Read code list for personality disorder in Supplementary material.

Word count: 3,997

For peer review only

Table 1 Baseline characteristics of study cohort

	All (n = 46210)		Recorded SMI (n = 9335)		No recorded SMI (n = 36875)	
	**Mean**	**(sd)**	**Mean**	**(sd)**	**Mean**	**(sd)**
**Age at study entry*, years**	42.65	(16.39)	43.37	(15.24)	42.47	(16.66)
	**N**	**(%)**	**N**	**(%)**	**N**	**(%)**
**Gender**						
Men	22682	(49.1)	4689	(50.2)	17993	(48.8)
Women	23528	(50.9)	4646	(49.8)	18882	(51.2)
**Age, years**						
0-15	384	(0.8)	6	(0.1)	378	(1.0)
15-19	1729	(3.7)	179	(1.9)	1550	(4.2)
20-24	4482	(9.7)	707	(7.6)	3775	(10.2)
25-29	5089	(11.0)	1004	(10.8)	4085	(11.1)
30-34	5312	(11.5)	1178	(12.6)	4134	(11.2)
35-39	5541	(12.0)	1291	(13.8)	4250	(11.5)
40-44	5251	(11.4)	1214	(13.0)	4037	(10.9)
45-49	4379	(9.5)	1002	(10.7)	3377	(9.2)
50-54	3802	(8.2)	783	(8.4)	3019	(8.2)
55-59	3053	(6.6)	596	(6.4)	2457	(6.7)
60-64	2335	(5.1)	469	(5.0)	1866	(5.1)
65-69	1657	(3.6)	311	(3.3)	1346	(3.7)
70-74	1152	(2.5)	214	(2.3)	938	(2.5)
75-100	2044	(4.4)	381	(4.1)	1663	(4.5)
**Townsend deprivation score**						
1 (Least deprived)	5523	(12.0)	893	(9.6)	4630	(12.6)
2	6700	(14.5)	1144	(12.3)	5556	(15.1)
3	9047	(19.6)	1762	(18.9)	7285	(19.8)
4	11654	(25.2)	2563	(27.5)	9091	(24.7)
5 (Most deprived)	11928	(25.8)	2693	(28.8)	9235	(25.0)
Missing	*1358*	*(2.9)*	*280*	*(3)*	*1078*	*(2.9)*
**Diagnostic category of PD**						
Paranoid	1988	(4.3)	981	(10.5)	1007	(2.7)
Schizoid	1346	(2.9)	565	(6.1)	781	(2.1)
Dissocial	2407	(5.2)	536	(5.7)	1871	(5.1)
Emotionally unstable	12300	(26.6)	2591	(27.8)	9709	(26.3)
Histrionic	1168	(2.5)	178	(1.9)	990	(2.7)
Anankastic	3648	(7.9)	347	(3.7)	3301	(9.0)
Anxious	501	(1.1)	62	(0.7)	439	(1.2)
Dependent	2705	(5.9)	411	(4.4)	2294	(6.2)
Other*	20147	(43.6)	3664	(39.3)	16483	(44.7)

For peer review only - http://bmjopen.bmj.com/site/about/guidelines.xhtml

**Recorded psychiatric comorbidity**						
Any SMI	9335	(20.2)	9335	(100)	0	(0)
Schizophrenia	*4105*	*(8.9)*	*4105*	*(44.0)*	*0*	*(0)*
Bipolar disorder	*2987*	*(6.5)*	*2987*	*(32.0)*	*0*	*(0)*
Other psychotic illness	*5071*	*(11.0)*	*5071*	*(54.3)*	*0*	*(0)*
Post-traumatic stress disorder	1468	(3.2)	306	(3.3)	1162	(3.2)
Eating disorder	2852	(6.2)	613	(6.6)	2239	(6.1)
Depression	34522	(74.7)	7116	(76.2)	27406	(74.3)
Anxiety	23595	(51.1)	5006	(53.6)	18589	(50.4)
Phobia	3279	(7.10)	666	(7.1)	2613	(7.1)
Hypochondria	416	(0.9)	117	(1.3)	299	(0.8)
Obsessive compulsive disorder	5333	(11.5)	831	(8.9)	4502	(12.2)
Other neurosis	1353	(2.9)	332	(3.6)	1021	(2.8)
**Recorded adverse childhood experience**						
Record	4742	(10.3)	946	(10.1)	3796	(10.3)
No record	41468	(89.7)	8389	(89.9)	33079	(89.7)

*Other PD includes: eccentric, haltlose, immature, narcissistic, masochistic, passive-aggressive, mixed and non-specific

For peer review only - http://bmjopen.bmj.com/site/about/guidelines.xhtml

Table 2 Proportions of individuals receiving at least one prescription for antipsychotics during follow-up

	Total	Prescribed antipsychotics, n (%)		Recorded SMI	Prescribed antipsychotics, n (%)		No recorded SMI	Prescribed antipsychotics, n (%)	
**All**	46210	15562	(33.7)	9335	6354	(68.1)	36875	9208	(25.0)
**Gender**									
Men	22682	6958	(30.7)	4689	3044	(64.9)	17993	3914	(21.8)
Women	23528	8604	(36.6)	4646	3310	(71.2)	18882	5294	(28.0)
**Age, years**									
0-15	384	26	(6.8)	6	4	(66.7)	378	22	(5.8)
15-19	1729	556	(32.2)	179	127	(70.9)	1550	429	(27.7)
20-24	4482	1543	(34.4)	707	450	(63.6)	3775	1093	(29.0)
25-29	5089	1866	(36.7)	1004	665	(66.2)	4085	1201	(29.4)
30-34	5312	2009	(37.8)	1178	835	(70.9)	4134	1174	(28.4)
35-39	5541	2068	(37.3)	1291	905	(70.1)	4250	1163	(27.4)
40-44	5251	1924	(36.6)	1214	840	(69.2)	4037	1084	(26.9)
45-49	4379	1503	(34.3)	1002	718	(71.7)	3377	785	(23.2)
50-54	3802	1169	(30.7)	783	524	(66.9)	3019	645	(21.4)
55-59	3053	816	(26.7)	596	421	(70.6)	2457	395	(16.1)
60-64	2335	586	(25.1)	469	308	(65.7)	1866	278	(14.9)
65-69	1657	420	(25.3)	311	190	(61.1)	1346	230	(17.1)
70-74	1152	337	(29.3)	214	128	(59.8)	938	209	(22.3)
75-100	2044	739	(36.2)	381	239	(62.7)	1663	500	(30.1)
**Townsend deprivation score**									
1 (Least deprived)	5523	1486	(26.9)	893	592	(66.3)	4630	894	(19.3)
2	6700	1932	(28.8)	1144	772	(67.5)	5556	1160	(20.9)
3	9047	2956	(32.7)	1762	1204	(68.3)	7285	1752	(24.0)

For peer review only - http://bmjopen.bmj.com/site/about/guidelines.xhtml

4	11654	4176	(35.8)	2563	1751	(68.3)	9091	2425	(26.7)
5 (Most deprived)	11928	4559	(38.2)	2693	1861	(69.1)	9235	2698	(29.2)
Missing	*1358*	*453*	*(33.4)*	*280*	*174*	*(62.1)*	*1078*	*279*	*(25.9)*
**Diagnostic category of PD**									
Paranoid	1988	1026	(51.6)	981	693	(70.6)	1007	333	(33.1)
Schizoid	1346	536	(39.8)	565	394	(69.7)	781	142	(18.2)
Dissocial	2407	763	(31.7)	536	337	(62.9)	1871	426	(22.8)
Emotionally unstable	12300	5573	(45.3)	2591	1850	(71.4)	9709	3723	(38.3)
Histrionic	1168	285	(24.4)	178	116	(65.2)	990	169	(17.1)
Anankastic	3648	700	(19.2)	347	223	(64.3)	3301	477	(14.5)
Anxious	501	130	(25.9)	62	42	(67.7)	439	88	(20.0)
Dependent	2705	686	(25.4)	411	303	(73.7)	2294	383	(16.7)
Other	20147	5863	(29.1)	3664	2396	(65.4)	16483	3467	(21.0)
**Psychiatric comorbidity**									
Any SMI	9335	6354	(68.1)	9335	6354	(68.1)	0	0	(.)
Schizophrenia	4105	2981	(72.6)	4105	2981	(72.6)	0	0	(.)
Bipolar disorder	2987	2044	(68.4)	2987	2044	(68.4)	0	0	(.)
Other psychotic illness	5071	3583	(70.7)	5071	3583	(70.7)	0	0	(.)
Post-traumatic stress disorder	1468	731	(49.8)	306	223	(72.9)	1162	508	(43.7)
Eating disorder	2852	1233	(43.2)	613	448	(73.1)	2239	785	(35.1)
Depression	34522	12698	(36.8)	7116	4979	(70.0)	27406	7719	(28.2)
Anxiety	23595	9201	(39.0)	5006	3601	(71.9)	18589	5600	(30.1)
Phobia	3279	1272	(38.8)	666	470	(70.6)	2613	802	(30.7)
Hypochondria	416	176	(42.3)	117	81	(69.2)	299	95	(31.8)
Obsessive compulsive disorder	5333	1517	(28.4)	831	560	(67.4)	4502	957	(21.3)
Other neurosis	1353	466	(34.4)	332	245	(73.8)	1021	221	(21.6)
No recorded psychiatric comorbidity	5763	874	(15.2)	0	0	(.)	5763	874	(15.2)

For peer review only

For peer review only - http://bmjopen.bmj.com/site/about/guidelines.xhtml

**Recorded adverse childhood experiences**									
Record	4742	1764	(37.2)	946	580	(61.3)	3796	1184	(31.2)
No record	41468	13798	(33.3)	8389	5774	(68.8)	33079	8024	(24.3)

For peer review only

For peer review only - http://bmjopen.bmj.com/site/about/guidelines.xhtml

Table 3 Rate ratios for receiving at least one antipsychotic prescription any time after first PD record

	Recorded SMI						No recorded SMI					
**Predictor**	**Univariable models**			**Multivariable models***			**Univariable models**			**Multivariable models***		
	**Rate ratio**	**95% CI**	**p**	**Rate ratio**	**95% CI**	**p**	**Rate ratio**	**95% CI**	**p**	**Rate ratio**	**95% CI**	**p**
**Gender**												
Men	1			1			1			1		
Women	1.29	(1.15, 1.45)	<0.001	1.17	(1.04, 1.32)	0.01	1.47	(1.34, 1.61)	<0.001	1.25	(1.16, 1.34)	<0.001
**Age, years**												
0-15	0.20	(0.05, 0.72)	0.01	0.18	(0.05, 0.58)	0.004	0.12	(0.08, 0.20)	<0.001	0.14	(0.08, 0.22)	<0.001
15-24	1.03	(0.85, 1.25)	0.8	0.89	(0.73, 1.08)	0.2	1.63	(1.45, 1.83)	<0.001	1.14	(1.01, 1.30)	0.04
25-34	1.21	(1.03, 1.43)	0.02	1.13	(0.96, 1.33)	0.1	1.38	(1.28, 1.50)	<0.001	1.17	(1.06, 1.30)	0.002
35-44	1			1			1			1		
45-54	0.99	(0.84, 1.17)	0.9	1.06	(0.89, 1.25)	0.5	0.68	(0.62, 0.73)	<0.001	0.78	(0.71, 0.85)	<0.001
55-64	0.77	(0.63, 0.95)	0.01	0.86	(0.70, 1.06)	0.2	0.41	(0.36, 0.46)	<0.001	0.53	(0.45, 0.61)	<0.001
65-74	0.68	(0.52, 0.89)	0.004	0.75	(0.57, 0.98)	0.04	0.55	(0.47, 0.65)	<0.001	0.74	(0.62, 0.89)	0.001
75-100	0.99	(0.76, 1.29)	0.9	1.10	(0.84, 1.44)	0.5	1.52	(1.31, 1.75)	<0.001	2.02	(1.75, 2.34)	<0.001
			<0.001†			*0.003†*			*<0.001†*			*<0.001†*
**Townsend deprivation score**												
1 (Least deprived)	0.75	(0.60, 0.93)	0.01	0.79	(0.63, 0.98)	0.03	0.46	(0.38, 0.56)	<0.001	0.56	(0.48, 0.66)	<0.001
2	0.83	(0.67, 1.03)	0.09	0.88	(0.71, 1.08)	0.2	0.53	(0.47, 0.60)	<0.001	0.64	(0.57, 0.71)	<0.001
3	0.85	(0.72, 1.00)	0.06	0.87	(0.74, 1.03)	0.1	0.70	(0.63, 0.76)	<0.001	0.78	(0.71, 0.84)	<0.001
4	0.89	(0.78, 1.03)	0.1	0.89	(0.78, 1.02)	0.1	0.87	(0.80, 0.94)	0.001	0.90	(0.83, 0.97)	0.007
5 (Most deprived)	1			1			1			1		
			0.08†			*0.2†*			*<0.001†*			*<0.001†*
**Diagnostic category of PD**												
Anankastic	0.32	(0.23, 0.44)	<0.001	0.35	(0.26, 0.48)	<0.001	0.14	(0.12, 0.16)	<0.001	0.19	(0.16, 0.21)	<0.001
Anxious	0.44	(0.25, 0.80)	0.006	0.47	(0.26, 0.85)	0.01	0.23	(0.15, 0.35)	<0.001	0.29	(0.20, 0.41)	<0.001
Emotionally unstable	1			1			1			1		
Dependent	0.57	(0.41, 0.78)	0.001	0.62	(0.44, 0.86)	0.004	0.17	(0.14, 0.21)	<0.001	0.22	(0.17, 0.27)	<0.001
Dissocial	0.60	(0.46, 0.80)	<0.001	0.66	(0.49, 0.88)	0.005	0.34	(0.28, 0.40)	<0.001	0.45	(0.39, 0.52)	<0.001
Histrionic	0.35	(0.24, 0.51)	<0.001	0.37	(0.25, 0.54)	<0.001	0.15	(0.13, 0.19)	<0.001	0.19	(0.15, 0.23)	<0.001
Other	0.54	(0.47, 0.62)	<0.001	0.57	(0.49, 0.67)	<0.001	0.27	(0.25, 0.30)	<0.001	0.33	(0.30, 0.36)	<0.001
Paranoid	0.68	(0.55, 0.83)	<0.001	0.72	(0.57, 0.91)	0.005	0.52	(0.44, 0.61)	<0.001	0.63	(0.53, 0.74)	<0.001
Schizoid	0.42	(0.32, 0.53)	<0.001	0.46	(0.35, 0.60)	<0.001	0.18	(0.14, 0.23)	<0.001	0.24	(0.18, 0.33)	<0.001

For peer review only - http://bmjopen.bmj.com/site/about/guidelines.xhtml

For peer review only

			<0.001 †			*<0.001* †			*<0.001* †			*<0.001* †
**Recorded adverse childhood experiences**												
Record	1			1			1			1		
No record	1.10	(0.92, 1.31)	0.3	0.93	(0.78, 1.10)	0.4	2.32	(2.03, 2.65)	<0.001	1.41	(1.28, 1.56)	<0.001

From Poisson regression, with generalised estimating equations to account for potential clustering of patients in general practices. *Adjusted for all the other characteristics considered [†]P-values in italics indicate significance of categorical variables as a whole, as opposed to the individual categories.

For peer review only

Fig 1 Percentage of cohort prescribed antipsychotics in each calendar year*

*Percentage of cohort prescribed antipsychotics in each calendar year = 100 x (number of people from cohort present in that calendar year with at least one antipsychotic prescription in that year)/(number of people from cohort present in that calendar year)

For peer review only

Reference List

(1) Tyrer P, Reed GM, Crawford MJ. Classification, assessment, prevalence, and effect of personality disorder. Lancet 2015 Feb 21;385(9969):717-26.

(2) Samuels J. Personality disorders: epidemiology and public health issues. Int Rev Psychiatry 2011 Jun;23(3):223-33.

(3) Moran P, Jenkins R, Tylee A, Blizard R, Mann A. The prevalence of personality disorder among UK primary care attenders. Acta Psychiatr Scand 2000 Jul;102(1):52-7.

(4) Bateman AW, Gunderson J, Mulder R. Treatment of personality disorder. Lancet 2015 Feb 21;385(9969):735-43.

(5) Kendall T, Pilling S, Tyrer P, Duggan C, Burbeck R, Meader N, et al. Borderline and antisocial personality disorders: summary of NICE guidance. BMJ 2009 Jan 28;338:b93.

(6) Gunderson JG. Clinical practice. Borderline personality disorder. N Engl J Med 2011 May 26;364(21):2037-42.

(7) Practice guideline for the treatment of patients with borderline personality disorder. American Psychiatric Association. Am J Psychiatry 2001 Oct;158(10 Suppl):1-52.

(8) Lieb K, Vollm B, Rucker G, Timmer A, Stoffers JM. Pharmacotherapy for borderline personality disorder: Cochrane systematic review of randomised trials. Br J Psychiatry 2010 Jan;196(1):4-12.

(9) Paton C, Crawford MJ, Bhatti SF, Patel MX, Barnes TR. The use of psychotropic medication in patients with emotionally unstable personality disorder under the care of UK mental health services. J Clin Psychiatry 2015 Apr;76(4):e512-e518.

(10) Newcomer JW. Antipsychotic medications: metabolic and cardiovascular risk. J Clin Psychiatry 2007;68 Suppl 4:8-13.

(11) Porter C, Palmier-Claus J, Branitsky A, Mansell W, Warwick H, Varese F. Childhood adversity and borderline personality disorder: a meta-analysis. Acta Psychiatr Scand 2020 Jan;141(1):6-20.

(12) Cummins I. The Impact of Austerity on Mental Health Service Provision: A UK Perspective. Int J Environ Res Public Health 2018 Jun 1;15(6).

(13) Blak BT, Thompson M, Dattani H, Bourke A. Generalisability of The Health Improvement Network (THIN) database: demographics, chronic disease prevalence and mortality rates. Inform Prim Care 2011;19(4):251-5.

(14) Chisholm J. The Read clinical classification. BMJ 1990 Apr 28;300(6732):1092.

For peer review only

(15) Dave S, Petersen I. Creating medical and drug code lists to identify cases in primary care databases. Pharmacoepidemiol Drug Saf 2009 Aug;18(8):704-7.

(16) Maguire A, Blak BT, Thompson M. The importance of defining periods of complete mortality reporting for research using automated data from primary care. Pharmacoepidemiol Drug Saf 2009 Jan;18(1):76-83.

(17) Horsfall L, Walters K, Petersen I. Identifying periods of acceptable computer usage in primary care research databases. Pharmacoepidemiol Drug Saf 2013 Jan;22(1):64-9.

(18) Townsend P. Deprivation. Journal of Social Policy 1987 Apr;16(2):125-46.

(19) Woodman J, Freemantle N, Allister J, de LS, Gilbert R, Petersen I. Variation in recorded child maltreatment concerns in UK primary care records: a cohort study using The Health Improvement Network (THIN) database. PLoS One 2012;7(11):e49808.

(20) Nose M, Cipriani A, Biancosino B, Grassi L, Barbui C. Efficacy of pharmacotherapy against core traits of borderline personality disorder: meta-analysis of randomized controlled trials. Int Clin Psychopharmacol 2006 Nov;21(6):345-53.

(21) Vita A, De PL, Sacchetti E. Antipsychotics, antidepressants, anticonvulsants, and placebo on the symptom dimensions of borderline personality disorder: a meta-analysis of randomized controlled and open-label trials. J Clin Psychopharmacol 2011 Oct;31(5):613-24.

(22) Anda RF, Brown DW, Felitti VJ, Bremner JD, Dube SR, Giles WH. Adverse childhood experiences and prescribed psychotropic medications in adults. Am J Prev Med 2007 May;32(5):389-94.

(23) Hardoon S, Hayes JF, Blackburn R, Petersen I, Walters K, Nazareth I, et al. Recording of severe mental illness in United Kingdom primary care, 2000-2010. PLoS One 2013;8(12):e82365.

(24) Marston L, Nazareth I, Petersen I, Walters K, Osborn DP. Prescribing of antipsychotics in UK primary care: a cohort study. BMJ Open 2014 Dec 18;4(12):e006135.

(25) Martinez C, Jones RW, Rietbrock S. Trends in the prevalence of antipsychotic drug use among patients with Alzheimer's disease and other dementias including those treated with antidementia drugs in the community in the UK: a cohort study. BMJ Open 2013 Jan 7;3(1).

(26) Nemeroff CB, Heim CM, Thase ME, Klein DN, Rush AJ, Schatzberg AF, et al. Differential responses to psychotherapy versus pharmacotherapy in patients with chronic forms of major depression and childhood trauma. Proc Natl Acad Sci U S A 2003 Nov 25;100(24):14293-6.

For peer review only - http://bmjopen.bmj.com/site/about/guidelines.xhtml

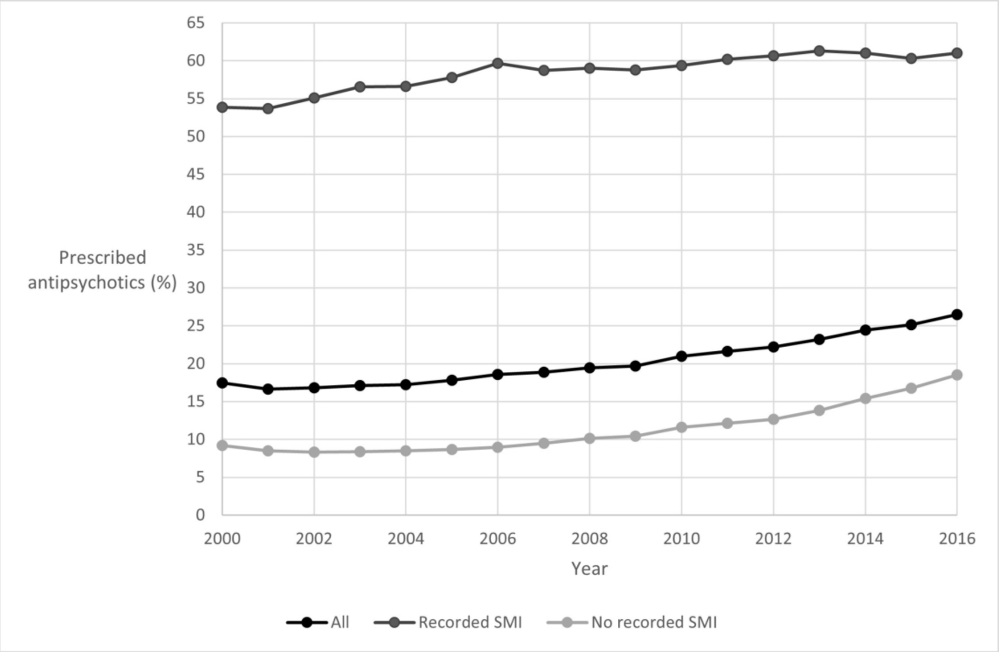

173x113mm (300 x 300 DPI)

Prescribing of antipsychotics among people with recorded Personality Disorder in primary care: a retrospective nationwide cohort study using The Health Improvement Network primary care database

Sarah L Hardoon[1], Joe F Hayes[1,2], Essi Viding[3], Eamon McCrory[3,4], Kate Walters[5], David PJ Osborn[1,2]

1 Division of Psychiatry, UCL, UK

2 Camden and Islington NHS Foundation Trust, UK

3 Division of Psychology and Language Sciences, UCL, UK

4 Anna Freud National Centre for Children and Families, UK

5 Department of Primary Care & Population Health, UCL, UK

For peer review only

Supplementary material

Appendix

Appendix Table 1 Read codes for personality disorder

Read code	Description	Diagnostic category of PD	N*	%
E21..00	Personality disorders	other or non-specific	14321	30.99
E21y200	Borderline personality disorder	emotionally unstable	4926	10.66
E21z.00	Personality disorder NOS	other or non-specific	4676	10.12
E215200	Emotionally unstable personality	emotionally unstable	3477	7.52
E214100	Obsessional personality	anankastic	2564	5.55
Eu60300	[X]Emotionally unstable personality disorder	emotionally unstable	1988	4.30
E210.00	Paranoid personality disorder	paranoid	1760	3.81
E216.00	Inadequate personality disorder	dependent	1282	2.77
E212.00	Schizoid personality disorder	schizoid	998	2.16
E216.13	Labile personality	dependent	752	1.63
E21z.11	Psychopathic personality	dissocial	722	1.56
E21y500	Immature personality disorder	other or non-specific	605	1.31
E213.00	Explosive personality disorder	emotionally unstable	555	1.20
E215.11	Hysterical personality disorders	histrionic	531	1.15
E217.00	Antisocial or sociopathic personality disorder	dissocial	455	0.98
Eu60200	[X]Dissocial personality disorder	dissocial	411	0.89
Eu60z00	[X]Personality disorder, unspecified	other or non-specific	374	0.81
E215.00	Histrionic personality disorders	histrionic	369	0.80
E21y711	Neurotic personality	other or non-specific	357	0.77
E21yz00	Other personality disorder NOS	other or non-specific	352	0.76
Eu60600	[X]Anxious [avoidant] personality disorder	anxious	337	0.73
Eu60212	[X]Antisocial personality disorder	dissocial	326	0.71
Eu60512	[X]Obsessional personality disorder	anankastic	301	0.65
E214.00	Compulsive personality disorders	anankastic	249	0.54
Eu61.00	[X]Mixed and other personality disorders	other or non-specific	239	0.52
E216.12	Dependent personality	dependent	238	0.51
Eu60700	[X]Dependent personality disorder	dependent	229	0.50
E21y700	Psychoneurotic personality disorder	other or non-specific	210	0.45
Eu60513	[X]Obsessive-compulsive personality disorder	anankastic	206	0.45
Eu60311	[X]Aggressive personality disorder	emotionally unstable	205	0.44
E21y.00	Other personality disorders	other or non-specific	204	0.44
Eu60312	[X]Borderline personality disorder	emotionally unstable	176	0.38

Eu60214	[X]Psychopathic personality disorder	dissocial	158	0.34
E21y100	Avoidant personality disorder	anxious	145	0.31
E212z00	Schizoid personality disorder NOS	schizoid	126	0.27
Eu60411	[X]Hysterical personality disorder	histrionic	123	0.27
Eu60100	[X]Schizoid personality disorder	schizoid	122	0.26
E21y000	Narcissistic personality disorder	other or non-specific	111	0.24
Eu60000	[X]Paranoid personality disorder	paranoid	110	0.24
Eu60215	[X]Sociopathic personality disorder	dissocial	110	0.24
E214000	Anankastic personality	anankastic	106	0.23
E21..11	Neurotic personality disorder	other or non-specific	80	0.17
E21y400	Eccentric personality disorder	other or non-specific	64	0.14
Eu60500	[X]Anankastic personality disorder	anankastic	57	0.12
E214z00	Compulsive personality disorder NOS	anankastic	52	0.11
Eu60712	[X]Inadequate personality disorder	dependent	39	0.08
Eu60400	[X]Histrionic personality disorder	histrionic	37	0.08
E215z00	Histrionic personality disorder NOS	histrionic	35	0.08
Eu60y13	[X]Immature personality disorder	other or non-specific	35	0.08
Eu60014	[X]Sensitive paranoid personality disorder	paranoid	33	0.07
Eu60511	[X]Compulsive personality disorder	anankastic	27	0.06
Eu60.00	[X]Specific personality disorders	other or non-specific	27	0.06
E212000	Unspecified schizoid personality disorder	schizoid	26	0.06
Eu60z12	[X]Pathological personality NOS	other or non-specific	22	0.05
Eu60y00	[X]Other specific personality disorders	other or non-specific	21	0.05
E215000	Unspecified histrionic personality disorder	histrionic	20	0.04
E216.11	Asthenic personality	dependent	20	0.04
E21y300	Passive-aggressive personality disorder	other or non-specific	20	0.04
Eu60y16	[X]Psychoneurotic personality disorder	other or non-specific	17	0.04
E214.11	Anancastic personality	anankastic	11	0.02
Eu60713	[X]Passive personality disorder	dependent	11	0.02
Eu60313	[X]Explosive personality disorder	emotionally unstable	10	0.02
Eu60z11	[X]Character neurosis NOS	other or non-specific	10	0.02
Eu60711	[X]Asthenic personality disorder	dependent	9	0.02
Eu60y14	[X]Narcissistic personality disorder	other or non-specific	8	0.02
Eu60y11	[X]Eccentric personality disorder	other or non-specific	6	0.01
Eu60714	[X]Self defeating personality disorder	dependent	4	0.01
Eu60213	[X]Asocial personality disorder	dissocial	3	0.01
E21y600	Masochistic personality disorder	other or non-specific	3	0.01
E217.11	Amoral personality	dissocial	2	<0.01
E215300	Psychoinfantile personality	histrionic	1	<0.01
Eu60412	[X]Psychoinfantile personality disorder	histrionic	1	<0.01
Eu60y12	[X]Haltlose type personality disorder	other or non-specific	1	<0.01
E210.11	Fanatic personality	paranoid	0	0
Eu60011	[X]Expansive paranoid personality disorder	paranoid	0	0
Eu60012	[X]Fanatic paranoid personality disorder	paranoid	0	0
Eu60013	[X]Querulant personality disorder	paranoid	0	0
Eu60211	[X]Amoral personality disorder	dissocial	0	0
Eu60y15	[X]Passive-aggressive personality disorder	other or non-specific	0	0

*N = number in cohort with the Read code as their most recent specific Read code for PD, unless individual had only non-specific Read codes, in which case, instead it is the most recent non-specific Read code.

For peer review only

Appendix Table 2 Types of antipsychotics prescribed

	All prescribed antipsychotics, n = 15562		Prescribed antipsychotics, SMI, n = 6354		All prescribed antipsychotics, no SMI, n = 9208	
	N	(%)	N	(%)	N	(%)
Atypical	12007	(77.2)	5370	(84.5)	6637	(72.1)
Typical	6688	(43.0)	2756	(43.4)	3932	(42.7)
QUETIAPINE	5819	(37.4)	2162	(34.0)	3657	(39.7)
OLANZAPINE	4269	(27.4)	2344	(36.9)	1925	(20.9)
RISPERIDONE	3399	(21.8)	1739	(27.4)	1660	(18.0)
CHLORPROMAZINE	2426	(15.6)	968	(15.2)	1458	(15.8)
ARIPIPRAZOLE	1728	(11.1)	1015	(16.0)	713	(7.7)
HALOPERIDOL	1298	(8.3)	606	(9.5)	692	(7.5)
FLUPENTIXOL	1041	(6.7)	459	(7.2)	582	(6.3)
TRIFLUOPERAZINE	951	(6.1)	407	(6.4)	544	(5.9)
AMISULPRIDE	854	(5.5)	549	(8.6)	305	(3.3)
PROMAZINE	666	(4.3)	215	(3.4)	451	(4.9)
THIORIDAZINE	560	(3.6)	212	(3.3)	348	(3.8)
SULPIRIDE	472	(3.0)	303	(4.8)	169	(1.8)
ZUCLOPENTHIXOL	365	(2.3)	256	(4.0)	109	(1.2)
LEVOMEPROMAZINE	354	(2.3)	90	(1.4)	264	(2.9)
PERICYAZINE	245	(1.6)	77	(1.2)	168	(1.8)
CLOZAPINE	146	(0.9)	129	(2.0)	17	(0.2)
FLUPHENAZINE	122	(0.8)	77	(1.2)	45	(0.5)
PERPHENAZINE	65	(0.4)	19	(0.3)	46	(0.5)
PIPOTIAZINE	43	(0.3)	36	(0.6)	7	(0.1)
PALIPERIDONE	41	(0.3)	33	(0.5)	8	(0.1)
DROPERIDOL	23	(0.1)	18	(0.3)	5	(0.1)
PIMOZIDE	19	(0.1)	15	(0.2)	4	(0.1)
BENPERIDOL	14	(0.1)	7	(0.1)	7	(0.1)
LURASIDONE	7	(0.1)	6	(0.1)	1	(0.1)
ASENAPINE	5	(0.1)	3	(0.1)	2	(0.1)
ZOTEPINE	5	(0.1)	4	(0.1)	1	(0.1)
LOXAPINE	2	(0.1)	1	(0.1)	1	(0.1)
SERTINDOLE	2	(0.1)	2	(0.1)	0	(0.1)
OXYPERTINE	1	(0.1)	1	(0.1)	0	(0.1)

Individuals may receive more than one type of antipsychotic so percentages add up to more than 100%. Other antipsychotics considered but which were not prescribed to anyone in the cohort: Chlorprothixene, Fluspirilene, Remoxipride, Thiopropazate, Thioproperazine, Trifluperidol.

For peer review only

Appendix Table 3 Proportions of cohort with repeat prescriptions and at least 6 months, 1 year, 3 years and 5 years continuous antipsychotic use

	Total present for time period	With length of antipsychotic use, n (%)		Total with recorded SMI present for time period	With length of antipsychotic use, n (%)		Total with no recorded SMI present for time period	With length of antipsychotic use, n (%)	
**At least two prescriptions ≤ 84 days apart**	46210	13898	(30.1)	9335	5973	(64.0)	36875	7925	(21.5)
**≥6 months continuous use**	40850	11906	(29.1)	8281	5358	(64.7)	32569	6548	(20.1)
**≥1 year continuous use**	35794	9588	(26.8)	7218	4591	(63.6)	28576	4997	(17.5)
**≥3 year continuous use**	24081	5492	(22.8)	4741	2954	(62.3)	19340	2538	(13.1)
**≥5 year continuous use**	17588	3529	(20.1)	3385	2044	(60.4)	14203	1485	(10.5)

For peer review only

STROBE Statement—Checklist of items that should be included in reports of ***cohort studies***

	Item No	Recommendation	Page No
**Title and abstract**	1	(*a*) Indicate the study's design with a commonly used term in the title or the abstract<br>(*b*) Provide in the abstract an informative and balanced summary of what was done and what was found	1
**Introduction**			
Background/rationale	2	Explain the scientific background and rationale for the investigation being reported	2-3
Objectives	3	State specific objectives, including any prespecified hypotheses	3
**Methods**			
Study design	4	Present key elements of study design early in the paper	3
Setting	5	Describe the setting, locations, and relevant dates, including periods of recruitment, exposure, follow-up, and data collection	3-4
Participants	6	(*a*) Give the eligibility criteria, and the sources and methods of selection of participants. Describe methods of follow-up<br>(*b*) For matched studies, give matching criteria and number of exposed and unexposed	3-4
Variables	7	Clearly define all outcomes, exposures, predictors, potential confounders, and effect modifiers. Give diagnostic criteria, if applicable	4-5
Data sources/ measurement	8*	For each variable of interest, give sources of data and details of methods of assessment (measurement). Describe comparability of assessment methods if there is more than one group	4-5
Bias	9	Describe any efforts to address potential sources of bias	3,5
Study size	10	Explain how the study size was arrived at	3
Quantitative variables	11	Explain how quantitative variables were handled in the analyses. If applicable, describe which groupings were chosen and why	5
Statistical methods	12	(*a*) Describe all statistical methods, including those used to control for confounding<br>(*b*) Describe any methods used to examine subgroups and interactions<br>(*c*) Explain how missing data were addressed<br>(*d*) If applicable, explain how loss to follow-up was addressed<br>(*e*) Describe any sensitivity analyses	5
**Results**			
Participants	13*	(a) Report numbers of individuals at each stage of study—eg numbers potentially eligible, examined for eligibility, confirmed eligible, included in the study, completing follow-up, and analysed<br>(b) Give reasons for non-participation at each stage<br>(c) Consider use of a flow diagram	5
Descriptive data	14*	(a) Give characteristics of study participants (eg demographic, clinical, social) and information on exposures and potential confounders<br>(b) Indicate number of participants with missing data for each variable of interest<br>(c) Summarise follow-up time (eg, average and total amount)	5-6, Table 1

For peer review only

For peer review only - http://bmjopen.bmj.com/site/about/guidelines.xhtml

Outcome data	15*	Report numbers of outcome events or summary measures over time	6, Table 2

For peer review only

For peer review only - http://bmjopen.bmj.com/site/about/guidelines.xhtml

Main results	16	(*a*) Give unadjusted estimates and, if applicable, confounder-adjusted estimates and their precision (eg, 95% confidence interval). Make clear which confounders were adjusted for and why they were included<br>(*b*) Report category boundaries when continuous variables were categorized<br>(*c*) If relevant, consider translating estimates of relative risk into absolute risk for a meaningful time period	6, Table 3
Other analyses	17	Report other analyses done—eg analyses of subgroups and interactions, and sensitivity analyses	6-7
**Discussion**			
Key results	18	Summarise key results with reference to study objectives	7
Limitations	19	Discuss limitations of the study, taking into account sources of potential bias or imprecision. Discuss both direction and magnitude of any potential bias	8
Interpretation	20	Give a cautious overall interpretation of results considering objectives, limitations, multiplicity of analyses, results from similar studies, and other relevant evidence	8-9
Generalisability	21	Discuss the generalisability (external validity) of the study results	8
**Other information**			
Funding	22	Give the source of funding and the role of the funders for the present study and, if applicable, for the original study on which the present article is based	10

*Give information separately for exposed and unexposed groups.

Note: An Explanation and Elaboration article discusses each checklist item and gives methodological background and published examples of transparent reporting. The STROBE checklist is best used in conjunction with this article (freely available on the Web sites of PLoS Medicine at http://www.plosmedicine.org/, Annals of Internal Medicine at http://www.annals.org/, and Epidemiology at http://www.epidem.com/). Information on the STROBE Initiative is available at http://www.strobe-statement.org.

For peer review only

For peer review only - http://bmjopen.bmj.com/site/about/guidelines.xhtml

BMJ Open

Prescribing of antipsychotics among people with recorded Personality Disorder in primary care: a retrospective nationwide cohort study using The Health Improvement Network primary care database

Journal:	*BMJ Open*
Manuscript ID	bmjopen-2021-053943.R1
Article Type:	Original research
Date Submitted by the Author:	20-Nov-2021
Complete List of Authors:	Hardoon, Sarah; UCL, Division of Psychiatry<br>Hayes, Joseph; UCL<br>Viding, Essi; UCL<br>McCrory, Eamon; UCL<br>Walters, Kate; UCL<br>Osborn, David; UCL
**Primary Subject Heading**:	Mental health
Secondary Subject Heading:	General practice / Family practice
Keywords:	Personality disorders < PSYCHIATRY, PRIMARY CARE, THERAPEUTICS

SCHOLARONE™
Manuscripts

BMJ

I, the Submitting Author has the right to grant and does grant on behalf of all authors of the Work (as defined in the below author licence), an exclusive licence and/or a non-exclusive licence for contributions from authors who are: i) UK Crown employees; ii) where BMJ has agreed a CC-BY licence shall apply, and/or iii) in accordance with the terms applicable for US Federal Government officers or employees acting as part of their official duties; on a worldwide, perpetual, irrevocable, royalty-free basis to BMJ Publishing Group Ltd ("BMJ") its licensees and where the relevant Journal is co-owned by BMJ to the co-owners of the Journal, to publish the Work in this journal and any other BMJ products and to exploit all rights, as set out in our licence.

The Submitting Author accepts and understands that any supply made under these terms is made by BMJ to the Submitting Author unless you are acting as an employee on behalf of your employer or a postgraduate student of an affiliated institution which is paying any applicable article publishing charge ("APC") for Open Access articles. Where the Submitting Author wishes to make the Work available on an Open Access basis (and intends to pay the relevant APC), the terms of reuse of such Open Access shall be governed by a Creative Commons licence – details of these licences and which Creative Commons licence will apply to this Work are set out in our licence referred to above.

Other than as permitted in any relevant BMJ Author's Self Archiving Policies, I confirm this Work has not been accepted for publication elsewhere, is not being considered for publication elsewhere and does not duplicate material already published. I confirm all authors consent to publication of this Work and authorise the granting of this licence.

For peer review only

Prescribing of antipsychotics among people with recorded Personality Disorder in primary care: a retrospective nationwide cohort study using The Health Improvement Network primary care database

Sarah L Hardoon[1], Joe F Hayes[1,2], Essi Viding[3], Eamon McCrory[3,4], Kate Walters[5], David PJ Osborn[1,2*]

1 Division of Psychiatry, UCL, UK

2 Camden and Islington NHS Foundation Trust, UK

3 Division of Psychology and Language Sciences, UCL, UK

4 Anna Freud National Centre for Children and Families, UK

5 Department of Primary Care & Population Health, UCL, UK

*Corresponding author; email: d.osborn@ucl.ac.uk

Abstract

Objectives To investigate the extent of antipsychotic prescribing to people with recorded personality disorder (PD) in United Kingdom (UK) primary care and factors associated with such prescribing.

Design Retrospective cohort study

Setting General practices contributing to The Health Improvement Network UK-wide primary care database, 1 January 2000 – 31 December 2016

Participants 46,210 people registered with participating general practices who had a record of PD in their general practice notes. 1,358 (2.9%) people with missing deprivation information were excluded from regression analyses; no other missing data.

Main outcome measures Prescriptions for antipsychotics in general practice records and length of time in receipt of antipsychotic prescriptions

Results Of 46,210 people with recorded PD, 15,562 (34%) were ever prescribed antipsychotics. Among the subgroup of 36,875 people with recorded PD, but no recorded severe mental illness (SMI), 9,208 (25%) were prescribed antipsychotics; prescribing was lower in less deprived areas (adjusted rate ratio (aRR) comparing least to most deprived quintile: 0.56, 95% CI 0.48-0.66, p<0.001), was higher in females (aRR:1.25, 95% CI 1.16-1.34, p<0.001), and with a history of adverse childhood experiences (aRR:1.44, 95% CI 1.28-1.56, p<0.001). Median time prescribed antipsychotics was 605 days (IQR 197-1639 days). Prescribing frequency has increased over time.

Conclusions Contrary to current UK guidelines, antipsychotics are frequently and increasingly prescribed for extended periods to people with recorded PD, but with no history of SMI. An urgent review of clinical practice is warranted, including the effectiveness of such prescribing and the need to monitor for adverse effects, including metabolic complications.

For peer review only - http://bmjopen.bmj.com/site/about/guidelines.xhtml

Article summary: Strengths and limitations of this study

- In the present report we identified a very large UK-wide cohort of >46,000 people with a recorded personality disorder diagnosis, enabling, for the first time, a detailed UK-wide exploration of prescribing practices in personality disorder (PD) over an 18-year period from 2000 to 2016.
- The cohort was derived from The Health Improvement Network primary care database which has been shown to be socially and demographically representative of wider UK general practice.
- The study benefits from complete records on the outcome (antipsychotics prescribed in primary care, including the type of antipsychotic), as all prescriptions are automatically computerised.
- Potential limitations include limited information on alternative treatment options, such as psychological therapy, and that it is not possible to ascertain how the PD diagnoses were arrived at or to verify them.

Introduction

The core features of personality disorder (PD) diagnoses are pervasive patterns of behaviour and maladaptive traits leading to substantial personal distress, social dysfunction and disruption to others, often beginning in early adult life(1). Prevalence of PD in the community is estimated to be 6-10%, but estimates for PD vary globally and by clinical setting(2), with limited research regarding the prevalence in primary care settings(3). There is limited evidence on the effectiveness of antipsychotic pharmacotherapy in PD. Results from randomised controlled trials mainly focus on emotionally unstable PD (EUPD) populations, but the findings are limited by short term follow-up, small sample sizes and variability in outcome measures (4). In the United Kingdom (UK), treatment guidelines (from the National Institute for Health and Care Excellence (NICE)) exist for EUPD and dissocial PD. For both diagnoses it is recommended that “drug treatment should not be used”, apart from short-term sedating medication during crises in EUPD(5). This is echoed by guidelines for EUPD from Australia’s National Health and Medical Research Council(6). Conversely, the American Psychiatric Association recommends targeted pharmacotherapy for PD: antidepressant medication for affective instability, mood stabilisers for impulsive aggression and antipsychotics for cognitive-perceptive disturbance(7). Cochrane reviews find no evidence for the efficacy of antidepressant medication, but some evidence that mood stabilisers may reduce affective dysregulation and impulsive–aggression and that antipsychotic medication may improve cognitive–perceptual symptoms and affective dysregulation in PD(8). Despite this mixed evidence, and counter to the NICE guidelines, there is some limited data suggesting that psychotropic prescribing for PD is occurring in the UK mental health service setting(9). There is an urgent need to ascertain the wider extent and duration of psychotropic prescribing in PD. There is a particular concern regarding the prescribing of antipsychotics given their adverse event profile, including metabolic and cardiovascular risks(10). Further, the characteristics of individuals prescribed psychotropic medications need to be determined to understand who may be most likely to receive these medications. For example, females are more likely than males to be diagnosed with borderline or

For peer review only - http://bmjopen.bmj.com/site/about/guidelines.xhtml

EUPD and there is a high prevalence of adverse childhood experiences among those with EUPD(11). Yet it is not known whether patients of particular gender, PD diagnosis or life history are more likely to receive antipsychotic medication. Time trends in antipsychotic prescribing for PD have not been examined and may be important, as UK mental health services and social care provision have been negatively affected by economic austerity(12). This study aimed to determine how frequently antipsychotics are prescribed for people with a diagnosis of PD in UK primary care as well as factors associated with this prescribing. The three main aims of the study were: 1) To quantify the prevalence and duration of antipsychotic prescribing among patients with a record of PD, particularly those without any diagnostic record of severe mental illness (SMI); 2) To determine whether gender, age, area level deprivation, mental health comorbidities, category of PD diagnosis or a recorded history of adverse childhood experiences affect the likelihood of being prescribed antipsychotics; 3) To examine time trends in prescribing of antipsychotics.

Methods

Study design

Retrospective nationwide cohort study

Data source

We used data from The Health Improvement Network (THIN) primary care database, which is a clinical database comprising, at time of analysis, computerised anonymised patient records retrieved from 744 member general practices across the UK, corresponding to approximately 6% of the UK population(13). Patient records are longitudinal, including all information recorded by the general practitioner (GP) from when the patient registered with the member practice until the date the practice last contributed data to THIN or the patient left the practice. Records for a patient are linked via an anonymised patient identifier.

The database includes all diagnoses, symptoms and other health data (blood tests, health indicators) recorded by the GP in the patient's computerised records, as well as all prescriptions issued. Diagnoses, symptoms and other health information in THIN are coded using the Read code clinical classification system(14). The database has been shown to be socially and demographically representative of the UK general practice population(13).

Study population

From the THIN database population we identified the smaller study population (our PD cohort) comprising people registered with a participating THIN general practice for at least some part of the period 2000-2016, who had a record in their general practice notes at any time during or prior to this period of a diagnosis of PD (specific or non-specific), identified via the presence of a Read code indicative of a PD diagnosis. A list of relevant Read codes was developed using established search techniques(15) and with reference to ICD-10 chapters F60-F61. The diagnostic list was verified by a clinician (JFH). The complete Read code list is provided in the Appendix (Supplementary material, Appendix Table 1). All permanently registered patients were included from the date the general practice reached previously defined, established data quality thresholds: acceptable mortality rate date(16) and acceptable computer usage date(17).

PD Read codes were categorised in line with current versions of the International Classification of Diseases (ICD-10) and the Diagnostic and Statistical Manual of Mental Disorders (DSM-5) as:

For peer review only

paranoid, schizoid, dissocial, EUPD (including borderline), histrionic, anankastic, anxious/avoidant, dependent and other (chiefly comprising codes for non-specific PD, but also including diagnoses such as eccentric, haltlose, immature, narcissistic, masochistic, passive-aggressive and mixed). Where an individual had more than one type of specific PD record, the most recent record was assigned as their diagnosis.

Outcomes

The primary outcome was a prescription for antipsychotic medication in the individual's primary care records, any time after the initial PD diagnosis. We included all first generation and second generation antipsychotics in British National Formulary (BNF) chapters 4.2.1; 4.2.2 (listed in Supplementary material, Appendix Table 2).

It is not possible to ascertain from THIN records whether a prescription that was issued was actually dispensed or used by the individual. Therefore we also included an outcome of at least two consecutive prescriptions being issued fewer than 84 days apart as a likely indication that the first prescription had been used (corresponds to 3 x 28 days, a common period for which longer term medicines are issued in UK primary care).

We also determined the length in days of continuous antipsychotic treatment. A continuous treatment period was defined as repeat prescriptions for antipsychotics no more than 84 days apart. The length of a continuous treatment period was then calculated from the date of the first prescription in the period to date of last prescription + 84 days.

Follow-up period

PD cohort entry was defined as the latest date of: registration at the general practice, the established practice acceptable mortality rate date (16), the established practice acceptable computer usage date (17), date of first PD diagnosis record, or 1 January 2000. End of follow-up for an individual was the earliest of: date of death, date patient transferred out of practice, latest date practice contributed data to THIN, or 31 December 2016.

Covariates

Antipsychotic prescribing was explored according to the following sociodemographic characteristics: gender, age and Townsend score for area-level social deprivation for the area (postcode) in which the individual lived (grouped into quintiles)(18). The Townsend score incorporates four variables: percentage of adults unemployed, percentages non-car ownership, percentage non-home ownership and percentage overcrowded households.

We extracted data regarding comorbid mental health conditions defined as: severe mental illness (SMI, including schizophrenia, bipolar disorder, and other non-organic psychotic illness)(19), depression, anxiety, phobia, hypochondria, obsessive compulsive disorder, other neurosis, eating disorder, post-traumatic stress disorder. Each of these conditions were identified by the presence of a Read code at any time, using lists of Read codes developed through established techniques, with reference to relevant ICD chapters and verified by clinician (JFH).

The presence of recorded adverse childhood experiences was identified from records with Read codes specifically relating to: contact with social services, child protection procedures, child on at risk register, child-at-risk case conference, out-of-home care, or codes corresponding to items in the Centers for Disease Control and Prevention short adverse childhood experiences tool(20), that is; direct reference to childhood maltreatment (physical or emotional abuse or neglect), or direct

For peer review only - http://bmjopen.bmj.com/site/about/guidelines.xhtml

For peer review only

reference to household dysfunction (such as parental substance misuse). Codes included were either i) codes recorded in childhood (when individual <17 years) or ii) codes recorded during adulthood indicative of a history of the above in childhood. The code list was based on previous work examining recording of childhood maltreatment in THIN(21).

Patient and public involvement

A group of service users was established in conjunction with the McPin Foundation, called the Pathfinder Data Science Patient and Public Involvement (PPI) Group. Through regular meetings over the course of the study, members of the group were involved in the design, conduct and reporting plans for our research.

Statistical analysis

We first determined the percentages of people: i) receiving at least one antipsychotic prescription, ii) at least two antipsychotic prescriptions fewer than 84 days apart. We also determined percentages of people with a continuous treatment periods of at least i) six months, ii) one year, iii) three years, iv) five years. Percentages were computed for the whole sample and then by: gender, age (in 10 year age bands), Townsend deprivation quintile, diagnostic category of PD, recorded mental health comorbidity, and whether an individual had a recorded history of adverse childhood experiences. Analyses were stratified by whether patients ever had an additional recorded diagnosis of SMI. For the continuous treatment periods percentages calculations, the study sample was restricted to only individuals who had at least that length of follow-up in the database (six months, one year, three years or five years).

We then used univariable and multivariable Poisson regression, with follow-up time for individuals as an offset, to estimate rate ratios for antipsychotic prescribing, to take account of differing follow-up times for individuals in the study. Rates were computed as total people receiving a prescription/ total follow-up time for people in each covariate category. Regressions were stratified by ever receiving a diagnosis of SMI. Multilevel Poisson models were used, with individuals nested in general practices, to account for potential clustering of individuals in general practices and clustering of prescribing practices within general practices.

Time trends in antipsychotic prescribing were determined as yearly prevalence estimates of antipsychotic prescriptions: namely the number within the sample with at least one antipsychotic prescription in that year/ number of the sample present in the database that year.

We calculated the median and interquartile range of length of longest continuous treatment period for all antipsychotics and for individual agents. The numbers of individuals receiving at least one prescription for individual agents were also computed.

We carried out two sensitivity analyses using different definitions of adverse childhood experiences. In the first sensitivity analysis, we restricted the definition of adverse childhood experiences to a record (current or retrospective) of contact with social services before age 17 years. In the second sensitivity analysis, we only included people with primary care data available from childhood (from at least age 11 years), to enable capture of adverse childhood experiences as recorded during childhood, rather than historically.

Stata (version 14) was used for all analyses.

For peer review only - http://bmjopen.bmj.com/site/about/guidelines.xhtml

Results

Our PD cohort comprised 46,210 people who had a record of PD in their general practice notes. Median duration in the study was 3.2 years (IQR 1.1 to 8.0 years). Just over half the PD cohort were female (23,528, 51%), and the average age at study entry was 43 years (Table 1). The PD cohort had greater levels of deprivation relative to the general population, with 11,928 (26%) of people living in areas within the most deprived quintile, and 5,523 (12%) in the least deprived quintile. The most commonly recorded diagnostic category of PD was EUPD recorded among 12,300 people (27% of the PD cohort), followed by anankastic (3,648, 8%). However, 20,147 (44%) of the PD cohort had only non-specific PD records including primarily 14,321 (31%) with Read code E21..00 “Personality Disorders” and 4,676 (10%) with Read code E21z.00 “Personality Disorders NOS” (Supplementary material, Appendix Table 1). A record of ever being diagnosed with an SMI was present in 9,335 people (20% of the PD cohort), while 34,522 (75%) and 23,595 (51%) had records of depression and anxiety respectively. A history of adverse childhood experiences was recorded for 4,742 people (10%) in the PD cohort.

Prevalence of antipsychotic prescribing

One third of the PD cohort of people with recorded diagnosis of PD were prescribed antipsychotics at least once after their first PD record (15,562, 34%, Table 2). The majority of those prescribed antipsychotics (9,208, 59%) in the PD cohort had no record of SMI to explain the prescribing. Indeed, restricting the PD cohort to 36,875 individuals with recorded PD but no record of SMI, one quarter (9,208, 25%) had been prescribed antipsychotics

Percentages of people in the PD cohort prescribed antipsychotics by gender, age, deprivation, mental health comorbidity, type of PD and recorded history of adverse childhood experiences, are shown in Table 2. Among those with recorded PD but *without* recorded SMI, prescribing of antipsychotics was higher among females than males (5,294 out of 18,882, 28% versus 3,914 out of 17,993, 22%) and highest among those aged 20-34 years and over 75 years, compared to other age groups. Prescribing increased with deprivation, from 19% (894 out of 4,630) in the least deprived quintile to 29% (2,698 out of 9,235) in the most deprived quintile. There was some variation in prescribing according to diagnostic category of PD, with prescribing highest among those with record of EUPD (3,723 out of 9,709, 38%), followed by paranoid PD (333 out of 1,007, 33%). Those who had a recorded history of adverse childhood experiences in their GP record were more likely to be prescribed antipsychotics than those who did not (1,184 out of 3,796, 31% versus 8,024 out of 33,079, 24%).

Adjusted and unadjusted rate ratios comparing rates of prescribing of antipsychotics for different levels of the socio-demographic characteristics, are shown in Table 3. The differences in prescribing of antipsychotics by gender, age, and deprivation, type of PD and record of adverse childhood experience all remained significant after adjustment in the group with no additional record of SMI. The adjusted rate ratio (aRR) comparing women to men was 1.25 (95% CI 1.16 to 1.34, $p<0.001$). The aRR comparing those over 75 years to those aged 35-44 years was 2.02 (1.75 to 2.34, $p<0.001$). The aRR comparing the least to most deprived areas was 0.56 (0.48 to 0.66, $p<0.001$). Rates for all diagnostic categories of PD were statistically significantly lower than for EUPD with aRRs relative to EUPD of between 0.19 (0.15 to 0.23, $p<0.001$) for anankastic PD and 0.63 (0.53 to 0.74, $p<0.001$) for paranoid PD. The aRR comparing history to no history of adverse childhood experiences was 1.41 (1.28 to 1.56, $p<0.001$).

For peer review only - http://bmjopen.bmj.com/site/about/guidelines.xhtml

Time trends

Figure 1 presents for each calendar year, the percentage of people in the PD cohort prescribed at least one antipsychotic in that year. The percentage of people receiving prescriptions increased over time: Of 6,465 individuals who were present in the PD cohort in 2000, 1,129 (17%) received antipsychotics in that year. Of 10,399 individuals present in the PD cohort in 2016, 2,755 (26%) received antipsychotics (Fig 1). Among those without a record of SMI, prescribing increased from 9% (484 out of 5,268) in 2000 to 19% (1,567 out of 8,451) in 2016.

Duration of antipsychotic treatment in personality disorder

The median duration of continuous prescriptions of antipsychotics was 605 days (IQR 197 to 1639 days). Among those with no recorded SMI, the median duration of continuous treatment was 439 days (IQR 150 to 1218 days). For each of these results we only assessed the longest period of treatment for each individual.

Among the total PD cohort of 46,210 people, 13,898 (30%) received at least two consecutive antipsychotic prescriptions within 84 days, and substantial numbers were prescribed antipsychotics over at least six months (11,906 out of 40,850 present for at least six months, 29%), one year (9,588 out of 35,794, 27%), three years (5,492 out of 24,081, 23%) and five years (3,529 out of 17,588, 20%) (Supplementary material, Appendix table 3). Among the subgroup of individuals without recorded SMI, 22% (7,925 out of 36,875) received at least two consecutive prescriptions within 84 days, and many were prescribed antipsychotics for more than six months (6,548 out of 32,569, 20%), one year (4,997 out of 28,576, 18%), three years (2,538 out of 19,340, 13%) and five years (1,485 out of 14,203, 11%).

Individual antipsychotics agents

The most commonly prescribed antipsychotics in the PD cohort were Quetiapine (prescribed to 37%; 5,819 of 15,562 individuals in receipt of antipsychotics), Olanzapine (prescribed to 27%; 4,269) and Risperidone (22%; 3,399) (Supplementary material, Appendix table 2). Similarly, among those without any record of SMI the three most commonly prescribed antipsychotics were: Quetiapine (40%; 3,657 of 9,208 individuals in receipt of antipsychotics), Olanzapine (21%; 1,925) and Risperidone (18%; 1,660). Individuals may receive more than one type of antipsychotic agent so the percentages in Appendix table 2 add up to more than 100%.

Sensitivity analyses regarding the definition of adverse childhood experiences

We restricted the definition of adverse childhood experiences to a record occurring before age 17 years and a record of contact with social services. Of 1,345 people who met this definition, 417 (31%) were prescribed antipsychotics. The rate ratios for all predictors of receiving antipsychotics were very similar to those when using the broader definition of adverse childhood experiences. People who met this definition has an adjusted rate ratio for receiving antipsychotics of 1.45 (95% CI 1.22 to 1.71, p<0.001), compared to individuals without such a history in their health record.

In a second sensitivity analysis, we only included people with primary care data available from childhood (from at least age 11 years), to enable capture of adverse childhood experiences as recorded during childhood, rather than historically. There were 21,647 individuals with such data available (47% of our total PD cohort). Among these individuals 1,483 (7%) had records of social services contact from childhood, while 2,805 (13%) had records in childhood referring to adverse childhood experiences (including but not limited to social services contact), and 3,601 (17%) had records at any time referring to adverse childhood experiences (so including those with only

For peer review only - http://bmjopen.bmj.com/site/about/guidelines.xhtml

retrospective records). The proportion of individuals within each of these groups prescribed antipsychotics were 34% (for childhood social service contact), 36% (record of trauma recorded in childhood) and 37% (record of childhood trauma recorded at any time). The adjusted rate ratio comparing those with and without social services contact in childhood, again among those without recorded SMI was 1.36 (95% CI 1.15 to 1.60, p<0.001). In summary, there was a statistically significant higher frequency of antipsychotic prescribing for people with a record of adverse childhood experiences history, which remained despite more rigorous definitions of childhood trauma.

Discussion

Principal findings

In this large UK-wide cohort of over 46,000 people with a record of PD diagnosis in their general practice notes, we found that more than a third of people with PD were prescribed antipsychotics in UK primary care. Prescribing was common amongst people with PD even when there was no record of a diagnosis of SMI for which antipsychotics are usually indicated. These antipsychotic medications were prescribed for considerable lengths of time (frequently over a year) and prescribing has become more common over the period of our study to 2016. Among those with recorded PD but *without* recorded SMI, antipsychotic prescriptions were more common among women, individuals living in areas with greater deprivation, individuals with a record of EUPD or paranoid PD, and where the individual also had a history of adverse childhood experiences. The finding regarding adverse childhood experiences remained robust despite several analyses restricting the definition of adverse childhood experiences.

Comparison with other studies

To our knowledge this is the first study to investigate the pattern of antipsychotic prescribing among people with records of PD in UK primary care. Our findings suggest that while NICE guidance does not recommend long term prescribing of antipsychotics in treatment of PD, this practice is common in real life samples (5). There is some evidence that particular symptoms occurring within PD may be treated by antipsychotics: impulsivity, aggression, poor interpersonal relationships, global functioning and cognitive-perceptual symptoms are improved in meta-analyses of trials in EUPD(22;23). It is potentially understandable that clinicians would extrapolate these findings to the broader PD population in lieu of psychological treatments which may be harder to access for those with PD or may not be the patient's preference for treatment. However, in our study these antipsychotics are clearly being prescribed for considerable lengths of time and these treatments may not be reviewed or monitored appropriately, including for metabolic side effects.

We are not aware of any previous studies which examine antipsychotic medication according to history of adverse childhood experiences in people with PD. However, previous studies in SMI found that higher adverse childhood experience questionnaire scores are associated with higher antipsychotic prescribing, irrespective of diagnosis(24). Our findings are in line with this evidence from other diagnostic categories, but primary care data do not permit interrogation of the decision-making process underlying prescribing.

For peer review only - http://bmjopen.bmj.com/site/about/guidelines.xhtml

Strengths and weaknesses

The strengths of our study include the large size of the PD cohort, enabling precise estimates, and UK-wide coverage. While the THIN database pertains to approximately 6% of the UK population, previous studies have shown the THIN database to be socially and demographically representative of wider UK general practice(13), thus the results may be cautiously generalised beyond the THIN database population to the wider UK general practice population. The study also benefits from complete records on the outcome (antipsychotics prescribed in primary care, including the type of antipsychotic), as all prescriptions are automatically computerised. Antipsychotics administered in secondary care, including some depot injections and clozapine would not be captured; the numbers in receipt of antipsychotics could be even higher if medication received in secondary care was also considered. We were not able to extract information on alternative treatment options, such as psychological therapy. All individuals with any record in their general practice notes of PD were included in the PD cohort. It is not possible to ascertain how the PD diagnoses were arrived at or to verify them. Therefore, there may be people in the PD cohort misclassified as having PD and some people with these problems will not be coded in the database. Similarly, only cases of SMI and adverse childhood experiences recorded using Read codes are captured. However, a previous study found prevalence of SMI diagnosis in THIN to be consistent with other epidemiological findings, suggesting that under recording is minimal(19). It is likely that some adverse childhood experiences will not be captured in the THIN data, where either the individual has not told their GP or the GP has not recorded this history as a Read code. The impact would be to underestimate the rate ratio for antipsychotic prescribing comparing those with and without adverse childhood experiences. However, sensitivity analyses restricting to a tighter definition of childhood contact with social services and limiting the PD cohort to those with records before aged 18 years and before aged 11 years did not meaningfully influence our main findings. A further limitation is that the clusters of PD severity used in other international settings are not coded in UK primary care. Results were not broken down by type of antipsychotic (first or second generation). However, since second generation antipsychotics are far more commonly prescribed in the cohort (Appendix table), we would expect the patterns in prescribing of all antipsychotics to largely reflect patterns for second generation antipsychotics

Interpretation, implications, conclusions and future research

We found that a considerable proportion of people with a record of PD in primary care had been prescribed antipsychotics, even when there was no recorded alternative indication (SMI record). A number of individuals received these medications for an extended period of time (at least 5 years). This is potentially concerning as longer term prescribing of antipsychotics is counter to current UK NICE guidelines, which do not support routine use of antipsychotics to treat PD (in the absence of SMI), on account of the limited evidence regarding their effectiveness for PD(5). The people in our PD cohort receiving antipsychotics will be at risk of experiencing adverse side-effects from the drugs, possibly without any proven long term benefit of these agents for their mental health. They may develop adverse physical effects and yet may not receive appropriate physical health monitoring for weight gain and metabolic disturbance, because in the UK National Health Service (NHS) physical health monitoring is currently only targeted at people with SMI. The lack of formal monitoring and protocols for prescribing antipsychotics in PD could also put people at risk of adverse effects of polypharmacy (for example other psychotropic medications or medications for physical long term conditions).

Possible explanations for the use of antipsychotics in PD could include lack of availability or long delays in access to other evidence based psychological therapies or because previous therapy/ other

For peer review only - http://bmjopen.bmj.com/site/about/guidelines.xhtml

treatments have not helped. We were unable to determine receipt of psychological therapies for emotional dysregulation and other problems as these therapies are usually provided in secondary care and not well recorded in primary care databases such as THIN. The use of antipsychotics could reflect patient or practitioner preference, due to belief in the effectiveness of antipsychotics for PD or indeed diagnostic uncertainty where mood instability or transient psychotic symptoms are present. It may be that the prescriptions issued in primary care are frequently provided as follow-on prescribing, after an initial prescription by a secondary care psychiatrist, without clear guidance on when to stop prescribing the antipsychotic drugs. The increase in antipsychotic prescriptions over time could reflect the impact of longer waiting lists for psychological interventions during a period of economic austerity, as well as general trends in prescribing of antipsychotics that has been observed for other conditions, and increased reporting of adverse childhood experiences, which were associated with higher levels of prescribing(25).

There were variations in antipsychotic prescribing levels by socio-demographic characteristics. Notably, prescribing was higher in the more deprived areas, which could reflect poorer access to psychological therapies or higher levels of distress. Prescribing was also highest among those aged over 75 years, who would be particularly susceptible to the adverse metabolic effects of antipsychotics. Studies have previously shown antipsychotics being prescribed for dementia(26), despite also not recommended, which could explain some of the prescribing of antipsychotics in this age group.

The higher levels of prescribing in individuals with histories of adverse childhood experiences are not possible to explain fully with routine primary care data. People with records of adverse childhood experiences were more likely have a record of EUPD, compared with other PD types (almost half of all people with adverse childhood experiences, compared with one quarter of those without), and were living in more deprived areas, both of these characteristics which were also associated with higher levels of antipsychotic prescriptions in our PD cohort. However, the increased rate of prescribing among people with adverse childhood experiences was observed even in multivariable models. This pattern is of particular concern as it is likely those with adverse childhood experiences might benefit more from appropriate psychological interventions as opposed to long term medications(27).

Further investigations are warranted to understand the reasons for longer term prescribing of antipsychotics in PD in primary care, particularly among those groups with higher prescribing levels. Future work should identify ways to address this and to promote recommended care pathways. Investigations of antipsychotic prescribing according to PD severity clusters is also warranted. Further research is needed to investigate the short and longer term effectiveness of antipsychotics in PD and the potential for adverse events in this group as well as the delivery of monitoring of such effects in primary care. This work would inform future guidelines regarding treatments for people with PD, to ultimately ensure people with PD receive the most appropriate, safe and effective treatment.

Ethics statement: Anonymised data were used throughout the study provided by the data provider to UCL. Studies using The Health Improvement Network (THIN) database have had initial ethical approval from the NHS South-East Multicentre Research Ethics Committee, subject to prior independent scientific review. The Scientific Review Committee (IQVIA) approved the study protocol (SRC Reference Number: 17THIN072) prior to its undertaking.

For peer review only - http://bmjopen.bmj.com/site/about/guidelines.xhtml

Competing interests: None declared

Funding Statement: This work was supported by the Medical Research Council (grant number MC_PC_17216); and the Wellcome Trust (JFH, grant number 211085/Z/18/Z)

Acknowledgments: The Pathfinder Data Science PPI Group, led by Dan Robotham, from the McPin Foundation for their input and feedback on the study. DPJO and JFH are supported by the University College London Hospitals National Institute for Health Research (NIHR) Biomedical Research Centre and the NIHR North Thames Applied Research Collaboration. This funder had no role in study design, data collection, data analysis, data interpretation, or writing of the report. The views expressed in this article are those of the authors and not necessarily those of the NHS, the NIHR, or the Department of Health and Social Care.

Author contribution: SLH: formulation of research questions, study design, extraction and analysis of data, interpretation of data, writing of first draft of paper, critical revision of paper for important intellectual content, approval of final draft. JFH: formulation of research questions, study design, acquisition of data, interpretation of data, writing of first draft of paper, critical revision of paper for important intellectual content, approval of final draft. EV: formulation of research questions, study design, interpretation of data, critical revision of paper for important intellectual content, approval of final draft. EM: formulation of research questions, study design, interpretation of data, critical revision of paper for important intellectual content, approval of final draft. KW: interpretation of data, critical revision of paper for important intellectual content, approval of final draft. DPJO: formulation of research questions, study design, interpretation of data, critical revision of paper for important intellectual content, approval of final draft.

Data sharing statement: No data are available as no new data collected. Read code list for personality disorder in Supplementary material.

For peer review only - http://bmjopen.bmj.com/site/about/guidelines.xhtml

Table 1 Baseline characteristics of study cohort

	All (n = 46210)		Recorded SMI (n = 9335)		No recorded SMI (n = 36875)	
	**Mean**	**(sd)**	**Mean**	**(sd)**	**Mean**	**(sd)**
**Age at study entry*, years**	42.65	(16.39)	43.37	(15.24)	42.47	(16.66)
	**N**	**(%)**	**N**	**(%)**	**N**	**(%)**
**Gender**						
Men	22682	(49.1)	4689	(50.2)	17993	(48.8)
Women	23528	(50.9)	4646	(49.8)	18882	(51.2)
**Age, years**						
0-15	384	(0.8)	6	(0.1)	378	(1.0)
15-19	1729	(3.7)	179	(1.9)	1550	(4.2)
20-24	4482	(9.7)	707	(7.6)	3775	(10.2)
25-29	5089	(11.0)	1004	(10.8)	4085	(11.1)
30-34	5312	(11.5)	1178	(12.6)	4134	(11.2)
35-39	5541	(12.0)	1291	(13.8)	4250	(11.5)
40-44	5251	(11.4)	1214	(13.0)	4037	(10.9)
45-49	4379	(9.5)	1002	(10.7)	3377	(9.2)
50-54	3802	(8.2)	783	(8.4)	3019	(8.2)
55-59	3053	(6.6)	596	(6.4)	2457	(6.7)
60-64	2335	(5.1)	469	(5.0)	1866	(5.1)
65-69	1657	(3.6)	311	(3.3)	1346	(3.7)
70-74	1152	(2.5)	214	(2.3)	938	(2.5)
75-100	2044	(4.4)	381	(4.1)	1663	(4.5)
**Townsend deprivation score**						
1 (Least deprived)	5523	(12.0)	893	(9.6)	4630	(12.6)
2	6700	(14.5)	1144	(12.3)	5556	(15.1)
3	9047	(19.6)	1762	(18.9)	7285	(19.8)
4	11654	(25.2)	2563	(27.5)	9091	(24.7)
5 (Most deprived)	11928	(25.8)	2693	(28.8)	9235	(25.0)
Missing	*1358*	*(2.9)*	*280*	*(3)*	*1078*	*(2.9)*
**ICD-10 diagnostic category of PD**						
Paranoid	1988	(4.3)	981	(10.5)	1007	(2.7)
Schizoid	1346	(2.9)	565	(6.1)	781	(2.1)
Dissocial	2407	(5.2)	536	(5.7)	1871	(5.1)
Emotionally unstable	12300	(26.6)	2591	(27.8)	9709	(26.3)
Histrionic	1168	(2.5)	178	(1.9)	990	(2.7)
Anankastic	3648	(7.9)	347	(3.7)	3301	(9.0)
Anxious	501	(1.1)	62	(0.7)	439	(1.2)
Dependent	2705	(5.9)	411	(4.4)	2294	(6.2)
Other*	20147	(43.6)	3664	(39.3)	16483	(44.7)

For peer review only

**Recorded psychiatric comorbidity**						
Any SMI	9335	(20.2)	9335	(100)	0	(0)
Schizophrenia	*4105*	*(8.9)*	*4105*	*(44.0)*	*0*	*(0)*
Bipolar disorder	*2987*	*(6.5)*	*2987*	*(32.0)*	*0*	*(0)*
Other psychotic illness	*5071*	*(11.0)*	*5071*	*(54.3)*	*0*	*(0)*
Post-traumatic stress disorder	1468	(3.2)	306	(3.3)	1162	(3.2)
Eating disorder	2852	(6.2)	613	(6.6)	2239	(6.1)
Depression	34522	(74.7)	7116	(76.2)	27406	(74.3)
Anxiety	23595	(51.1)	5006	(53.6)	18589	(50.4)
Phobia	3279	(7.10)	666	(7.1)	2613	(7.1)
Hypochondria	416	(0.9)	117	(1.3)	299	(0.8)
Obsessive compulsive disorder	5333	(11.5)	831	(8.9)	4502	(12.2)
Other neurosis	1353	(2.9)	332	(3.6)	1021	(2.8)
**Recorded adverse childhood experience**						
No record	41468	(89.7)	8389	(89.9)	33079	(89.7)
Record	4742	(10.3)	946	(10.1)	3796	(10.3)

*Other PD includes: eccentric, haltlose, immature, narcissistic, masochistic, passive-aggressive, mixed and non-specific

For peer review only - http://bmjopen.bmj.com/site/about/guidelines.xhtml

Table 2 Proportions of individuals receiving at least one prescription for antipsychotics during follow-up

	Total	Prescribed antipsychotics, n	(%)	Recorded SMI	Prescribed antipsychotics, n	(%)	No recorded SMI	Prescribed antipsychotics, n	(%)
**All**	46210	15562	(33.7)	9335	6354	(68.1)	36875	9208	(25.0)
**Gender**									
Men	22682	6958	(30.7)	4689	3044	(64.9)	17993	3914	(21.8)
Women	23528	8604	(36.6)	4646	3310	(71.2)	18882	5294	(28.0)
**Age, years**									
0-15	384	26	(6.8)	6	4	(66.7)	378	22	(5.8)
15-19	1729	556	(32.2)	179	127	(70.9)	1550	429	(27.7)
20-24	4482	1543	(34.4)	707	450	(63.6)	3775	1093	(29.0)
25-29	5089	1866	(36.7)	1004	665	(66.2)	4085	1201	(29.4)
30-34	5312	2009	(37.8)	1178	835	(70.9)	4134	1174	(28.4)
35-39	5541	2068	(37.3)	1291	905	(70.1)	4250	1163	(27.4)
40-44	5251	1924	(36.6)	1214	840	(69.2)	4037	1084	(26.9)
45-49	4379	1503	(34.3)	1002	718	(71.7)	3377	785	(23.2)
50-54	3802	1169	(30.7)	783	524	(66.9)	3019	645	(21.4)
55-59	3053	816	(26.7)	596	421	(70.6)	2457	395	(16.1)
60-64	2335	586	(25.1)	469	308	(65.7)	1866	278	(14.9)
65-69	1657	420	(25.3)	311	190	(61.1)	1346	230	(17.1)
70-74	1152	337	(29.3)	214	128	(59.8)	938	209	(22.3)
75-100	2044	739	(36.2)	381	239	(62.7)	1663	500	(30.1)
**Townsend deprivation score**									
1 (Least deprived)	5523	1486	(26.9)	893	592	(66.3)	4630	894	(19.3)
2	6700	1932	(28.8)	1144	772	(67.5)	5556	1160	(20.9)
3	9047	2956	(32.7)	1762	1204	(68.3)	7285	1752	(24.0)

For peer review only - http://bmjopen.bmj.com/site/about/guidelines.xhtml

4	11654	4176	(35.8)	2563	1751	(68.3)	9091	2425	(26.7)
5 (Most deprived)	11928	4559	(38.2)	2693	1861	(69.1)	9235	2698	(29.2)
Missing	*1358*	*453*	*(33.4)*	*280*	*174*	*(62.1)*	*1078*	*279*	*(25.9)*
**ICD-10 diagnostic category of PD**									
Paranoid	1988	1026	(51.6)	981	693	(70.6)	1007	333	(33.1)
Schizoid	1346	536	(39.8)	565	394	(69.7)	781	142	(18.2)
Dissocial	2407	763	(31.7)	536	337	(62.9)	1871	426	(22.8)
Emotionally unstable	12300	5573	(45.3)	2591	1850	(71.4)	9709	3723	(38.3)
Histrionic	1168	285	(24.4)	178	116	(65.2)	990	169	(17.1)
Anankastic	3648	700	(19.2)	347	223	(64.3)	3301	477	(14.5)
Anxious	501	130	(25.9)	62	42	(67.7)	439	88	(20.0)
Dependent	2705	686	(25.4)	411	303	(73.7)	2294	383	(16.7)
Other	20147	5863	(29.1)	3664	2396	(65.4)	16483	3467	(21.0)
**Psychiatric comorbidity**									
Any SMI	9335	6354	(68.1)	9335	6354	(68.1)	0	0	(.)
Schizophrenia	4105	2981	(72.6)	4105	2981	(72.6)	0	0	(.)
Bipolar disorder	2987	2044	(68.4)	2987	2044	(68.4)	0	0	(.)
Other psychotic illness	5071	3583	(70.7)	5071	3583	(70.7)	0	0	(.)
Post-traumatic stress disorder	1468	731	(49.8)	306	223	(72.9)	1162	508	(43.7)
Eating disorder	2852	1233	(43.2)	613	448	(73.1)	2239	785	(35.1)
Depression	34522	12698	(36.8)	7116	4979	(70.0)	27406	7719	(28.2)
Anxiety	23595	9201	(39.0)	5006	3601	(71.9)	18589	5600	(30.1)
Phobia	3279	1272	(38.8)	666	470	(70.6)	2613	802	(30.7)
Hypochondria	416	176	(42.3)	117	81	(69.2)	299	95	(31.8)
Obsessive compulsive disorder	5333	1517	(28.4)	831	560	(67.4)	4502	957	(21.3)
Other neurosis	1353	466	(34.4)	332	245	(73.8)	1021	221	(21.6)
No recorded psychiatric comorbidity	5763	874	(15.2)	0	0	(.)	5763	874	(15.2)

For peer review only

For peer review only - http://bmjopen.bmj.com/site/about/guidelines.xhtml

**Recorded adverse childhood experiences**									
No record	41468	13798	(33.3)	8389	5774	(68.8)	33079	8024	(24.3)
Record	4742	1764	(37.2)	946	580	(61.3)	3796	1184	(31.2)

For peer review only

Table 3 Rate ratios for receiving at least one antipsychotic prescription any time after first PD record

	Recorded SMI						No recorded SMI					
**Predictor**	**Univariable models**			**Multivariable models***			**Univariable models**			**Multivariable models***		
	**Rate ratio**	**95% CI**	**p**	**Rate ratio**	**95% CI**	**p**	**Rate ratio**	**95% CI**	**p**	**Rate ratio**	**95% CI**	**p**
**Gender**												
Men	1			1			1			1		
Women	1.29	(1.15, 1.45)	<0.001	1.17	(1.04, 1.32)	0.01	1.47	(1.34, 1.61)	<0.001	1.25	(1.16, 1.34)	<0.001
**Age, years**												
0-15	0.20	(0.05, 0.72)	0.01	0.18	(0.05, 0.58)	0.004	0.12	(0.08, 0.20)	<0.001	0.14	(0.08, 0.22)	<0.001
15-24	1.03	(0.85, 1.25)	0.8	0.89	(0.73, 1.08)	0.2	1.63	(1.45, 1.83)	<0.001	1.14	(1.01, 1.30)	0.04
25-34	1.21	(1.03, 1.43)	0.02	1.13	(0.96, 1.33)	0.1	1.38	(1.28, 1.50)	<0.001	1.17	(1.06, 1.30)	0.002
35-44	1			1			1			1		
45-54	0.99	(0.84, 1.17)	0.9	1.06	(0.89, 1.25)	0.5	0.68	(0.62, 0.73)	<0.001	0.78	(0.71, 0.85)	<0.001
55-64	0.77	(0.63, 0.95)	0.01	0.86	(0.70, 1.06)	0.2	0.41	(0.36, 0.46)	<0.001	0.53	(0.45, 0.61)	<0.001
65-74	0.68	(0.52, 0.89)	0.004	0.75	(0.57, 0.98)	0.04	0.55	(0.47, 0.65)	<0.001	0.74	(0.62, 0.89)	0.001
75-100	0.99	(0.76, 1.29)	0.9	1.10	(0.84, 1.44)	0.5	1.52	(1.31, 1.75)	<0.001	2.02	(1.75, 2.34)	<0.001
			<0.001†			*0.003†*			*<0.001†*			*<0.001†*
**Townsend deprivation score**												
1 (Least deprived)	0.75	(0.60, 0.93)	0.01	0.79	(0.63, 0.98)	0.03	0.46	(0.38, 0.56)	<0.001	0.56	(0.48, 0.66)	<0.001
2	0.83	(0.67, 1.03)	0.09	0.88	(0.71, 1.08)	0.2	0.53	(0.47, 0.60)	<0.001	0.64	(0.57, 0.71)	<0.001
3	0.85	(0.72, 1.00)	0.06	0.87	(0.74, 1.03)	0.1	0.70	(0.63, 0.76)	<0.001	0.78	(0.71, 0.84)	<0.001
4	0.89	(0.78, 1.03)	0.1	0.89	(0.78, 1.02)	0.1	0.87	(0.80, 0.94)	0.001	0.90	(0.83, 0.97)	0.007
5 (Most deprived)	1			1			1			1		
			0.08†			*0.2†*			*<0.001†*			*<0.0*

For peer review only - http://bmjopen.bmj.com/site/about/guidelines.xhtml

For peer review only

												01†
**ICD-10 diagnostic category of PD**												
Anankastic	0.32	(0.23, 0.44)	<0.001	0.35	(0.26, 0.48)	<0.001	0.14	(0.12, 0.16)	<0.001	0.19	(0.16, 0.21)	<0.001
Anxious	0.44	(0.25, 0.80)	0.006	0.47	(0.26, 0.85)	0.01	0.23	(0.15, 0.35)	<0.001	0.29	(0.20, 0.41)	<0.001
Emotionally unstable	1			1			1			1		
Dependent	0.57	(0.41, 0.78)	0.001	0.62	(0.44, 0.86)	0.004	0.17	(0.14, 0.21)	<0.001	0.22	(0.17, 0.27)	<0.001
Dissocial	0.60	(0.46, 0.80)	<0.001	0.66	(0.49, 0.88)	0.005	0.34	(0.28, 0.40)	<0.001	0.45	(0.39, 0.52)	<0.001
Histrionic	0.35	(0.24, 0.51)	<0.001	0.37	(0.25, 0.54)	<0.001	0.15	(0.13, 0.19)	<0.001	0.19	(0.15, 0.23)	<0.001
Other	0.54	(0.47, 0.62)	<0.001	0.57	(0.49, 0.67)	<0.001	0.27	(0.25, 0.30)	<0.001	0.33	(0.30, 0.36)	<0.001
Paranoid	0.68	(0.55, 0.83)	<0.001	0.72	(0.57, 0.91)	0.005	0.52	(0.44, 0.61)	<0.001	0.63	(0.53, 0.74)	<0.001
Schizoid	0.42	(0.32, 0.53)	<0.001	0.46	(0.35, 0.60)	<0.001	0.18	(0.14, 0.23)	<0.001	0.24	(0.18, 0.33)	<0.001
			<0.001†			*<0.001*†			*<0.001*†			*<0.001*†
**Recorded adverse childhood experiences**												
No Record	1			1			1			1		
Record	1.10	(0.92, 1.31)	0.3	0.93	(0.78, 1.10)	0.4	2.32	(2.03, 2.65)	<0.001	1.41	(1.28, 1.56)	<0.001

From Poisson regression, with generalised estimating equations to account for potential clustering of patients in general practices. *Adjusted for all the other characteristics considered †P-values in italics indicate significance of categorical variables as a whole, as opposed to the individual categories.

For peer review only

Fig 1 Percentage of cohort prescribed antipsychotics in each calendar year*

*Percentage of cohort prescribed antipsychotics in each calendar year = 100 x (number of people from cohort present in that calendar year with at least one antipsychotic prescription in that year)/(number of people from cohort present in that calendar year)

For peer review only

Reference List

(1) Tyrer P, Reed GM, Crawford MJ. Classification, assessment, prevalence, and effect of personality disorder. Lancet 2015 Feb 21;385(9969):717-26.

(2) Samuels J. Personality disorders: epidemiology and public health issues. Int Rev Psychiatry 2011 Jun;23(3):223-33.

(3) Moran P, Jenkins R, Tylee A, Blizard R, Mann A. The prevalence of personality disorder among UK primary care attenders. Acta Psychiatr Scand 2000 Jul;102(1):52-7.

(4) Bateman AW, Gunderson J, Mulder R. Treatment of personality disorder. Lancet 2015 Feb 21;385(9969):735-43.

(5) Kendall T, Pilling S, Tyrer P, Duggan C, Burbeck R, Meader N, et al. Borderline and antisocial personality disorders: summary of NICE guidance. BMJ 2009 Jan 28;338:b93.

(6) Gunderson JG. Clinical practice. Borderline personality disorder. N Engl J Med 2011 May 26;364(21):2037-42.

(7) Practice guideline for the treatment of patients with borderline personality disorder. American Psychiatric Association. Am J Psychiatry 2001 Oct;158(10 Suppl):1-52.

(8) Lieb K, Vollm B, Rucker G, Timmer A, Stoffers JM. Pharmacotherapy for borderline personality disorder: Cochrane systematic review of randomised trials. Br J Psychiatry 2010 Jan;196(1):4-12.

(9) Paton C, Crawford MJ, Bhatti SF, Patel MX, Barnes TR. The use of psychotropic medication in patients with emotionally unstable personality disorder under the care of UK mental health services. J Clin Psychiatry 2015 Apr;76(4):e512-e518.

(10) Newcomer JW. Antipsychotic medications: metabolic and cardiovascular risk. J Clin Psychiatry 2007;68 Suppl 4:8-13.

(11) Porter C, Palmier-Claus J, Branitsky A, Mansell W, Warwick H, Varese F. Childhood adversity and borderline personality disorder: a meta-analysis. Acta Psychiatr Scand 2020 Jan;141(1):6-20.

(12) Cummins I. The Impact of Austerity on Mental Health Service Provision: A UK Perspective. Int J Environ Res Public Health 2018 Jun 1;15(6).

(13) Blak BT, Thompson M, Dattani H, Bourke A. Generalisability of The Health Improvement Network (THIN) database: demographics, chronic disease prevalence and mortality rates. Inform Prim Care 2011;19(4):251-5.

(14) Chisholm J. The Read clinical classification. BMJ 1990 Apr 28;300(6732):1092.

For peer review only - http://bmjopen.bmj.com/site/about/guidelines.xhtml

(15) Dave S, Petersen I. Creating medical and drug code lists to identify cases in primary care databases. Pharmacoepidemiol Drug Saf 2009 Aug;18(8):704-7.

(16) Maguire A, Blak BT, Thompson M. The importance of defining periods of complete mortality reporting for research using automated data from primary care. Pharmacoepidemiol Drug Saf 2009 Jan;18(1):76-83.

(17) Horsfall L, Walters K, Petersen I. Identifying periods of acceptable computer usage in primary care research databases. Pharmacoepidemiol Drug Saf 2013 Jan;22(1):64-9.

(18) Townsend P. Deprivation. Journal of Social Policy 1987 Apr;16(2):125-46.

(19) Hardoon S, Hayes JF, Blackburn R, Petersen I, Walters K, Nazareth I, et al. Recording of severe mental illness in United Kingdom primary care, 2000-2010. PLoS One 2013;8(12):e82365.

(20) Anda RF, Butchart A, Felitti VJ, Brown DW. Building a framework for global surveillance of the public health implications of adverse childhood experiences. Am J Prev Med 2010;39(1):93-8.

(21) Woodman J, Freemantle N, Allister J, de LS, Gilbert R, Petersen I. Variation in recorded child maltreatment concerns in UK primary care records: a cohort study using The Health Improvement Network (THIN) database. PLoS One 2012;7(11):e49808.

(22) Nose M, Cipriani A, Biancosino B, Grassi L, Barbui C. Efficacy of pharmacotherapy against core traits of borderline personality disorder: meta-analysis of randomized controlled trials. Int Clin Psychopharmacol 2006 Nov;21(6):345-53.

(23) Vita A, De PL, Sacchetti E. Antipsychotics, antidepressants, anticonvulsants, and placebo on the symptom dimensions of borderline personality disorder: a meta-analysis of randomized controlled and open-label trials. J Clin Psychopharmacol 2011 Oct;31(5):613-24.

(24) Anda RF, Brown DW, Felitti VJ, Bremner JD, Dube SR, Giles WH. Adverse childhood experiences and prescribed psychotropic medications in adults. Am J Prev Med 2007 May;32(5):389-94.

(25) Marston L, Nazareth I, Petersen I, Walters K, Osborn DP. Prescribing of antipsychotics in UK primary care: a cohort study. BMJ Open 2014 Dec 18;4(12):e006135.

(26) Martinez C, Jones RW, Rietbrock S. Trends in the prevalence of antipsychotic drug use among patients with Alzheimer's disease and other dementias including those treated with antidementia drugs in the community in the UK: a cohort study. BMJ Open 2013 Jan 7;3(1).

(27) Nemeroff CB, Heim CM, Thase ME, Klein DN, Rush AJ, Schatzberg AF, et al. Differential responses to psychotherapy versus pharmacotherapy in patients with chronic forms of major depression and childhood trauma. Proc Natl Acad Sci U S A 2003 Nov 25;100(24):14293-6.

For peer review only - http://bmjopen.bmj.com/site/about/guidelines.xhtml

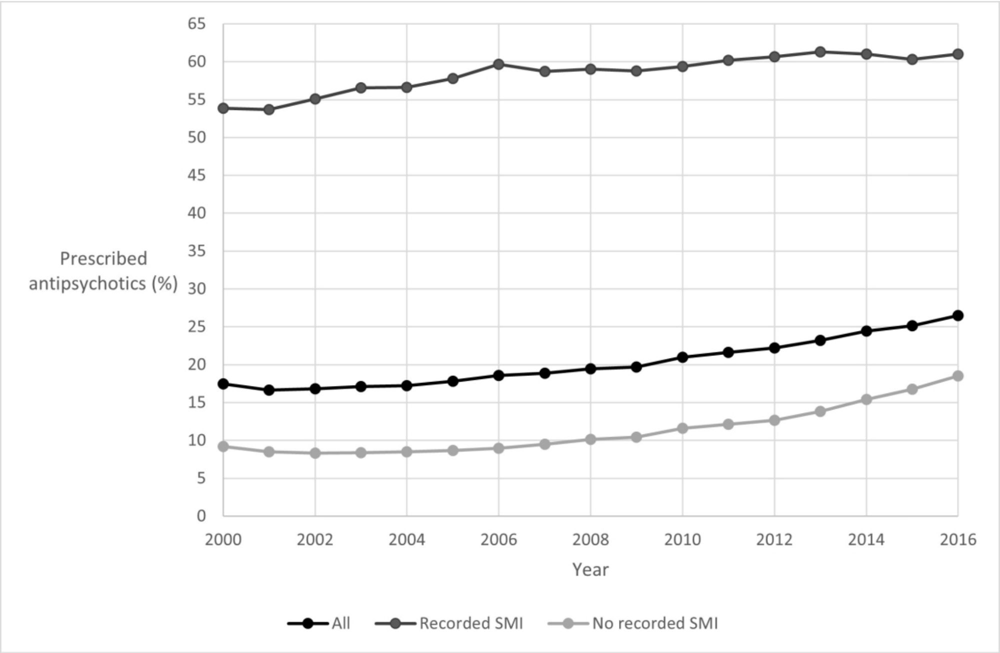

173x113mm (330 x 330 DPI)

Prescribing of antipsychotics among people with recorded Personality Disorder in primary care: a retrospective nationwide cohort study using The Health Improvement Network primary care database

Sarah L Hardoon[1], Joe F Hayes[1,2], Essi Viding[3], Eamon McCrory[3,4], Kate Walters[5], David PJ Osborn[1,2]

1 Division of Psychiatry, UCL, UK

2 Camden and Islington NHS Foundation Trust, UK

3 Division of Psychology and Language Sciences, UCL, UK

4 Anna Freud National Centre for Children and Families, UK

5 Department of Primary Care & Population Health, UCL, UK

Supplementary material

Appendix

Appendix Table 1 Read codes for personality disorder

Read code	Description	ICD-10 diagnostic category of PD	N*	%
E21..00	Personality disorders	other or non-specific	14321	30.99
E21y200	Borderline personality disorder	emotionally unstable	4926	10.66
E21z.00	Personality disorder NOS	other or non-specific	4676	10.12
E215200	Emotionally unstable personality	emotionally unstable	3477	7.52
E214100	Obsessional personality	anankastic	2564	5.55
Eu60300	[X]Emotionally unstable personality disorder	emotionally unstable	1988	4.30
E210.00	Paranoid personality disorder	paranoid	1760	3.81
E216.00	Inadequate personality disorder	dependent	1282	2.77
E212.00	Schizoid personality disorder	schizoid	998	2.16
E216.13	Labile personality	dependent	752	1.63
E21z.11	Psychopathic personality	dissocial	722	1.56
E21y500	Immature personality disorder	other or non-specific	605	1.31
E213.00	Explosive personality disorder	emotionally unstable	555	1.20
E215.11	Hysterical personality disorders	histrionic	531	1.15
E217.00	Antisocial or sociopathic personality disorder	dissocial	455	0.98
Eu60200	[X]Dissocial personality disorder	dissocial	411	0.89
Eu60z00	[X]Personality disorder, unspecified	other or non-specific	374	0.81
E215.00	Histrionic personality disorders	histrionic	369	0.80
E21y711	Neurotic personality	other or non-specific	357	0.77
E21yz00	Other personality disorder NOS	other or non-specific	352	0.76
Eu60600	[X]Anxious [avoidant] personality disorder	anxious	337	0.73
Eu60212	[X]Antisocial personality disorder	dissocial	326	0.71
Eu60512	[X]Obsessional personality disorder	anankastic	301	0.65
E214.00	Compulsive personality disorders	anankastic	249	0.54
Eu61.00	[X]Mixed and other personality disorders	other or non-specific	239	0.52
E216.12	Dependent personality	dependent	238	0.51
Eu60700	[X]Dependent personality disorder	dependent	229	0.50
E21y700	Psychoneurotic personality disorder	other or non-specific	210	0.45
Eu60513	[X]Obsessive-compulsive personality disorder	anankastic	206	0.45
Eu60311	[X]Aggressive personality disorder	emotionally unstable	205	0.44
E21y.00	Other personality disorders	other or non-specific	204	0.44
Eu60312	[X]Borderline personality disorder	emotionally unstable	176	0.38

For peer review only

Eu60214	[X]Psychopathic personality disorder	dissocial	158	0.34
E21y100	Avoidant personality disorder	anxious	145	0.31
E212z00	Schizoid personality disorder NOS	schizoid	126	0.27
Eu60411	[X]Hysterical personality disorder	histrionic	123	0.27
Eu60100	[X]Schizoid personality disorder	schizoid	122	0.26
E21y000	Narcissistic personality disorder	other or non-specific	111	0.24
Eu60000	[X]Paranoid personality disorder	paranoid	110	0.24
Eu60215	[X]Sociopathic personality disorder	dissocial	110	0.24
E214000	Anankastic personality	anankastic	106	0.23
E21..11	Neurotic personality disorder	other or non-specific	80	0.17
E21y400	Eccentric personality disorder	other or non-specific	64	0.14
Eu60500	[X]Anankastic personality disorder	anankastic	57	0.12
E214z00	Compulsive personality disorder NOS	anankastic	52	0.11
Eu60712	[X]Inadequate personality disorder	dependent	39	0.08
Eu60400	[X]Histrionic personality disorder	histrionic	37	0.08
E215z00	Histrionic personality disorder NOS	histrionic	35	0.08
Eu60y13	[X]Immature personality disorder	other or non-specific	35	0.08
Eu60014	[X]Sensitive paranoid personality disorder	paranoid	33	0.07
Eu60511	[X]Compulsive personality disorder	anankastic	27	0.06
Eu60.00	[X]Specific personality disorders	other or non-specific	27	0.06
E212000	Unspecified schizoid personality disorder	schizoid	26	0.06
Eu60z12	[X]Pathological personality NOS	other or non-specific	22	0.05
Eu60y00	[X]Other specific personality disorders	other or non-specific	21	0.05
E215000	Unspecified histrionic personality disorder	histrionic	20	0.04
E216.11	Asthenic personality	dependent	20	0.04
E21y300	Passive-aggressive personality disorder	other or non-specific	20	0.04
Eu60y16	[X]Psychoneurotic personality disorder	other or non-specific	17	0.04
E214.11	Anancastic personality	anankastic	11	0.02
Eu60713	[X]Passive personality disorder	dependent	11	0.02
Eu60313	[X]Explosive personality disorder	emotionally unstable	10	0.02
Eu60z11	[X]Character neurosis NOS	other or non-specific	10	0.02
Eu60711	[X]Asthenic personality disorder	dependent	9	0.02
Eu60y14	[X]Narcissistic personality disorder	other or non-specific	8	0.02
Eu60y11	[X]Eccentric personality disorder	other or non-specific	6	0.01
Eu60714	[X]Self defeating personality disorder	dependent	4	0.01
Eu60213	[X]Asocial personality disorder	dissocial	3	0.01
E21y600	Masochistic personality disorder	other or non-specific	3	0.01
E217.11	Amoral personality	dissocial	2	<0.01
E215300	Psychoinfantile personality	histrionic	1	<0.01
Eu60412	[X]Psychoinfantile personality disorder	histrionic	1	<0.01
Eu60y12	[X]Haltlose type personality disorder	other or non-specific	1	<0.01
E210.11	Fanatic personality	paranoid	0	0
Eu60011	[X]Expansive paranoid personality disorder	paranoid	0	0
Eu60012	[X]Fanatic paranoid personality disorder	paranoid	0	0
Eu60013	[X]Querulant personality disorder	paranoid	0	0
Eu60211	[X]Amoral personality disorder	dissocial	0	0
Eu60y15	[X]Passive-aggressive personality disorder	other or non-specific	0	0

*N = number in cohort with the Read code as their most recent specific Read code for PD, unless individual had only non-specific Read codes, in which case, instead it is the most recent non-specific Read code.

For peer review only

Appendix Table 2 Types of antipsychotics prescribed

	Typical (T) or Atypical (A)	All prescribed antipsychotics, n = 15562		Prescribed antipsychotics, SMI, n = 6354		All prescribed antipsychotics, no SMI, n = 9208	
		N	(%)	N	(%)	N	(%)
Atypical		12007	(77.2)	5370	(84.5)	6637	(72.1)
Typical		6688	(43.0)	2756	(43.4)	3932	(42.7)
QUETIAPINE	A	5819	(37.4)	2162	(34.0)	3657	(39.7)
OLANZAPINE	A	4269	(27.4)	2344	(36.9)	1925	(20.9)
RISPERIDONE	A	3399	(21.8)	1739	(27.4)	1660	(18.0)
CHLORPROMAZINE	T	2426	(15.6)	968	(15.2)	1458	(15.8)
ARIPIPRAZOLE	A	1728	(11.1)	1015	(16.0)	713	(7.7)
HALOPERIDOL	T	1298	(8.3)	606	(9.5)	692	(7.5)
FLUPENTIXOL	T	1041	(6.7)	459	(7.2)	582	(6.3)
TRIFLUOPERAZINE	T	951	(6.1)	407	(6.4)	544	(5.9)
AMISULPRIDE	A	854	(5.5)	549	(8.6)	305	(3.3)
PROMAZINE	T	666	(4.3)	215	(3.4)	451	(4.9)
THIORIDAZINE	T	560	(3.6)	212	(3.3)	348	(3.8)
SULPIRIDE	T	472	(3.0)	303	(4.8)	169	(1.8)
ZUCLOPENTHIXOL	T	365	(2.3)	256	(4.0)	109	(1.2)
LEVOMEPROMAZINE	T	354	(2.3)	90	(1.4)	264	(2.9)
PERICYAZINE	T	245	(1.6)	77	(1.2)	168	(1.8)
CLOZAPINE	A	146	(0.9)	129	(2.0)	17	(0.2)
FLUPHENAZINE	T	122	(0.8)	77	(1.2)	45	(0.5)
PERPHENAZINE	T	65	(0.4)	19	(0.3)	46	(0.5)
PIPOTIAZINE	T	43	(0.3)	36	(0.6)	7	(0.1)
PALIPERIDONE	T	41	(0.3)	33	(0.5)	8	(0.1)
DROPERIDOL	T	23	(0.1)	18	(0.3)	5	(0.1)
PIMOZIDE	T	19	(0.1)	15	(0.2)	4	(0.1)
BENPERIDOL	T	14	(0.1)	7	(0.1)	7	(0.1)
LURASIDONE	A	7	(0.1)	6	(0.1)	1	(0.1)
ASENAPINE	A	5	(0.1)	3	(0.1)	2	(0.1)
ZOTEPINE	A	5	(0.1)	4	(0.1)	1	(0.1)
LOXAPINE	T	2	(0.1)	1	(0.1)	1	(0.1)
SERTINDOLE	A	2	(0.1)	2	(0.1)	0	(0.1)
OXYPERTINE	T	1	(0.1)	1	(0.1)	0	(0.1)

Individuals may receive more than one type of antipsychotic so percentages add up to more than 100%. Other antipsychotics considered but which were not prescribed to anyone in the cohort: Chlorprothixene, Fluspirilene, Remoxipride, Thiopropazate, Thioproperazine, Trifluperidol.

For peer review only

Appendix Table 3 Proportions of cohort with repeat prescriptions and at least 6 months, 1 year, 3 years and 5 years continuous antipsychotic use

	Total present for time period	With length of antipsychotic use, n (%)		Total with recorded SMI present for time period	With length of antipsychotic use, n (%)		Total with no recorded SMI present for time period	With length of antipsychotic use, n (%)	
**At least two prescriptions ≤ 84 days apart**	46210	13898	(30.1)	9335	5973	(64.0)	36875	7925	(21.5)
**≥6 months continuous use**	40850	11906	(29.1)	8281	5358	(64.7)	32569	6548	(20.1)
**≥1 year continuous use**	35794	9588	(26.8)	7218	4591	(63.6)	28576	4997	(17.5)
**≥3 year continuous use**	24081	5492	(22.8)	4741	2954	(62.3)	19340	2538	(13.1)
**≥5 year continuous use**	17588	3529	(20.1)	3385	2044	(60.4)	14203	1485	(10.5)

For peer review only

STROBE Statement—Checklist of items that should be included in reports of ***cohort studies***

	Item No	Recommendation	Page No
**Title and abstract**	1	(*a*) Indicate the study's design with a commonly used term in the title or the abstract<br>(*b*) Provide in the abstract an informative and balanced summary of what was done and what was found	1
**Introduction**			
Background/rationale	2	Explain the scientific background and rationale for the investigation being reported	2-3
Objectives	3	State specific objectives, including any prespecified hypotheses	3
**Methods**			
Study design	4	Present key elements of study design early in the paper	3
Setting	5	Describe the setting, locations, and relevant dates, including periods of recruitment, exposure, follow-up, and data collection	3-4
Participants	6	(*a*) Give the eligibility criteria, and the sources and methods of selection of participants. Describe methods of follow-up<br>(*b*) For matched studies, give matching criteria and number of exposed and unexposed	3-4
Variables	7	Clearly define all outcomes, exposures, predictors, potential confounders, and effect modifiers. Give diagnostic criteria, if applicable	4-5
Data sources/ measurement	8*	For each variable of interest, give sources of data and details of methods of assessment (measurement). Describe comparability of assessment methods if there is more than one group	4-5
Bias	9	Describe any efforts to address potential sources of bias	3,5
Study size	10	Explain how the study size was arrived at	3
Quantitative variables	11	Explain how quantitative variables were handled in the analyses. If applicable, describe which groupings were chosen and why	5
Statistical methods	12	(*a*) Describe all statistical methods, including those used to control for confounding<br>(*b*) Describe any methods used to examine subgroups and interactions<br>(*c*) Explain how missing data were addressed<br>(*d*) If applicable, explain how loss to follow-up was addressed<br>(*e*) Describe any sensitivity analyses	5
**Results**			
Participants	13*	(a) Report numbers of individuals at each stage of study—eg numbers potentially eligible, examined for eligibility, confirmed eligible, included in the study, completing follow-up, and analysed<br>(b) Give reasons for non-participation at each stage<br>(c) Consider use of a flow diagram	5
Descriptive data	14*	(a) Give characteristics of study participants (eg demographic, clinical, social) and information on exposures and potential confounders<br>(b) Indicate number of participants with missing data for each variable of interest<br>(c) Summarise follow-up time (eg, average and total amount)	5-6, Table 1

For peer review only - http://bmjopen.bmj.com/site/about/guidelines.xhtml

Outcome data	15*	Report numbers of outcome events or summary measures over time	6, Table 2

For peer review only

For peer review only - http://bmjopen.bmj.com/site/about/guidelines.xhtml

Main results	16	(*a*) Give unadjusted estimates and, if applicable, confounder-adjusted estimates and their precision (eg, 95% confidence interval). Make clear which confounders were adjusted for and why they were included<br>(*b*) Report category boundaries when continuous variables were categorized<br>(*c*) If relevant, consider translating estimates of relative risk into absolute risk for a meaningful time period	6, Table 3
Other analyses	17	Report other analyses done—eg analyses of subgroups and interactions, and sensitivity analyses	6-7
**Discussion**			
Key results	18	Summarise key results with reference to study objectives	7
Limitations	19	Discuss limitations of the study, taking into account sources of potential bias or imprecision. Discuss both direction and magnitude of any potential bias	8
Interpretation	20	Give a cautious overall interpretation of results considering objectives, limitations, multiplicity of analyses, results from similar studies, and other relevant evidence	8-9
Generalisability	21	Discuss the generalisability (external validity) of the study results	8
**Other information**			
Funding	22	Give the source of funding and the role of the funders for the present study and, if applicable, for the original study on which the present article is based	10

*Give information separately for exposed and unexposed groups.

Note: An Explanation and Elaboration article discusses each checklist item and gives methodological background and published examples of transparent reporting. The STROBE checklist is best used in conjunction with this article (freely available on the Web sites of PLoS Medicine at http://www.plosmedicine.org/, Annals of Internal Medicine at http://www.annals.org/, and Epidemiology at http://www.epidem.com/). Information on the STROBE Initiative is available at http://www.strobe-statement.org.

For peer review only

For peer review only - http://bmjopen.bmj.com/site/about/guidelines.xhtml
